# Targeted deletion of c-kit in TECs attenuates UUO-induced renal fibrosis through NF-κB pathway inhibition

**DOI:** 10.1038/s41598-026-42540-w

**Published:** 2026-03-12

**Authors:** Zenghui Xing, Hongfeng Wang, Yaguang Zhou, Shengxin Chen, Bing Han, Yixuan Zhang, Shengchun Zheng, Yan Chen, Guangyan Cai, Lei Shen, Xiangmei Chen, Xuefeng Sun

**Affiliations:** 1 Senior Department of Nephrology, State Key Laboratory of Kidney Diseases, Chinese PLA General Hospital, National Clinical Research Center for Kidney Diseases, No. 28 Fuxing Road, Haidian District, Beijing, 100853 China; 2 Department of Dermatology, Hainan Hospital of PLA General Hospital, Sanya, 572013 Hainan Province China; 3 Department of Gastroenterology and Hepatology, The First Medical Center, Chinese PLA General Hospital, 28 Fuxing Road, Haidian District, Beijing, 100853 China; 4 Department of Nephrology, Tianjin Union Medical Center, The First Affiliated Hospital of Nankai University, Tianjin, 300121 China

**Keywords:** SCF/c-kit, UUO, RIF, TECs, NF-κB, Cell biology, Diseases, Nephrology

## Abstract

Renal interstitial fibrosis (RIF) is the cardinal pathological hallmark of chronic kidney disease (CKD). Although the stem-cell factor (SCF)/c-kit axis has been implicated in kidney fibrogenesis, its cell-specific role and downstream mechanism in tubular epithelial cells (TECs) remain undefined. We investigated whether TEC-derived SCF/c-kit signaling propels RIF after unilateral ureteral obstruction (UUO) via Nuclear Factor Kappa-Light-Chain-Enhancer of Activated B Cells (NF-κB) activation. Using the Cre-loxP system, a renal tubular epithelial cell-specific c-kit gene knockout mouse model (Ggt1-Cre c-kit^−/−^) was established. Renal interstitial fibrosis was induced in both C57BL/6J (WT) and Ggt1-Cre c-kit^−/−^ mice via UUO surgery. The extent of fibrosis was evaluated by assessing renal function indicators (serum creatinine and blood urea nitrogen), renal histopathology (PAS and Sirius red staining), and the expression of fibrosis markers (α-SMA, Vimentin, and Collagen I). Western blot and immunofluorescence techniques were employed to detect the expression of fibrosis markers, key proteins of the NF-κB pathway (p-p65, p65, p-IκBα, and IκBα), and inflammatory cytokines (Interleukin (IL)-6 and IL-1β). Primary renal tubular epithelial cells were isolated and cultured ex vivo, and treated with SCF and the NF-κB-specific inhibitor SC-75741 to evaluate the degree of cellular fibrosis and the activation status of the NF-κB pathway. Fourteen days after UUO, renal SCF and c-kit expression paralleled the rise in fibrotic markers. TEC‑specific deletion of c‑kit ameliorates the initial decline in renal function, attenuates tubular injury, reduces collagen deposition, and downregulates the expression of fibrotic proteins. Genetic deletion of c-kit significantly inhibits NF-κB phosphorylation and the expression of inflammatory cytokines IL-6 and IL-1β. In vitro, SCF provoked a fibrogenic phenotype in WT TECs that was abolished by either c-kit deletion or SC-75741. The SCF/c-kit signaling pathway in TECs promotes renal interstitial fibrosis in UUO mice via activation of the NF-κB pathway. Targeted inhibition of c-kit in TECs alleviates inflammatory responses and fibrosis, improves renal function, and may offer a novel therapeutic target for delaying the progression of CKD.

**Supplementary Information:**

The online version contains supplementary material available at 10.1038/s41598-026-42540-w.

## Introduction

The global prevalence of chronic kidney disease (CKD) has been increasing annually, with the total number of patients exceeding 850 million by 2022^1^. Renal interstitial fibrosis (RIF) represents a core pathological process in the progression of CKD, characterized by progressive decline in renal function and irreversible fibrotic changes in renal tissue. Therefore, in-depth investigation into the pathophysiology of renal interstitial fibrosis and the identification of effective therapeutic targets are of profound clinical significance for preventing the progression of kidney disease to CKD.

Stem cell factor (SCF, also known as Steel factor or kit ligand) is expressed in both membrane-bound and soluble isoforms by fibroblasts and endothelial cells throughout the body^2^. Its cognate receptor, c-kit (CD117), is a 976-amino-acid type-III receptor tyrosine kinase whose name reflects its ligand-binding specificity^3^. The SCF/c-kit axis governs the migration, differentiation, and proliferation of hematopoietic, germ-line, and melanocytic progenitors^4–6^ and has been implicated in the fibrotic remodeling of multiple organs^7–9^. Kidneys undergoing fibrosis are often accompanied by high expression of SCF and α-smooth muscle actin (α-SMA)^10^.

Mast-cell density is markedly increased in fibrotic kidneys, and SCF—recognized as the principal mast-cell agonist—drives their chemotaxis, adhesion, and proliferation^11,12^. This observation has fostered the view that SCF/c-kit signaling promotes renal interstitial fibrosis chiefly through mast-cell activation. However, tubular epithelial cells (TECs) themselves constitutively express both SCF and c-kit, and tubulointerstitial injury contributes more decisively than glomerular damage to the progression of CKD^13,14^. Therefore, identifying intervenable molecular targets within TECs holds profound clinical significance for delaying the progression of CKD.

Our previous study demonstrated that systemic c-kit gene knockout mice exhibited significant amelioration of renal fibrosis, suggesting the potential existence of a fibrosis regulatory mechanism independent of mast cells^15^. In addition to enhancing local inflammatory responses in renal tissue by activating mast cells, does SCF also directly act on c-kit in TECs, thereby promoting the occurrence and progression of RIF?

In this study, a novel TEC-specific c-kit gene knockout mouse model was established. Unilateral ureteral obstruction (UUO) was induced to RIF, allowing for an in-depth investigation into the potential mechanisms of SCF/c-kit signaling. This research aims to provide new therapeutic targets and directions for the treatment of CKD.

## Materials and methods

### Experimental animals and grouping

Generation of tubule-specific c-kit knockout mice via the cre-loxp system. Tubule-specific c-kit knockout mice were generated by crossing c-kit conditional gene-targeted mice (c-kit ^loxp/loxp^, Cyagen Biosciences, Beijing) with mice expressing the renal tubule epithelial cell-specific Ggt1-Cre recombinase (Cyagen Biosciences, Beijing). Offspring were genotyped, and homozygous mice with the genotype Ggt1-Cre; c-kit ^loxp/loxp^ were selected as the experimental group (hereafter referred to as the Ggt1-Cre c-kit^−/−^ group). Male C57BL/6J mice (SPF Biotechnology, Beijing) served as the wild-type control group (WT). All mice were bred and housed under specific pathogen-free (SPF) conditions with a 12-hour light/dark cycle, ad libitum access to food and water, a room temperature of 22–25 °C, and a relative humidity of 40–60%. UUO surgery was performed when the animals reached 8 weeks of age (weight 19–22 g), with six mice per group. All animal experiments were performed in accordance with the ARRIVE (Animal Research: Reporting of In Vivo Experiments) guidelines. The study was approved by the Animal Ethics Committee of the Chinese PLA General Hospital and Military Medical College(2022-X18-09). All methods were performed in accordance with the relevant guidelines and regulations.

### Genotyping by tail biopsy

A tail tip segment (approximately 0.5–2 mm) was excised from each mouse using alcohol-sterilized scissors and placed into a labeled 1.5 mL EP tube. Genomic DNA was extracted using a Rapid Genotyping Kit for Mice (Beijing Sunsharp Technology Co., Ltd. Cat No. C190801). The tissue was lysed to release DNA, and the supernatant was collected as the template for PCR amplification. Specific primers (designed and synthesized by Beijing Liuhe BGI Technology Co., Ltd. Table 1) were used to amplify the target regions. The PCR products were then separated and visualized via agarose gel electrophoresis.

**Table 1:** Primer Sequences for Genotyping by Tail Biopsy.

Primer set	Sequence (5’→3’)	Amplicon (bp)	Detects
Flox-F1	AATGATGTTCATCGAGCATGAGAG	188/247	WT/flox
Flox-R1	GAGACATGAACATTAAGGGATGCTG		
Ggt1-Cre-F	CATCACATCAG GCACCCCAGAA	428	Ggt1-Cre
Ggt1-Cre-R	GAACATCTTCAGGTTCTGCGGGA		

### Establishment of unilateral ureteral obstruction model and collection of renal and serum samples

Mice were anesthetized by intraperitoneal injection of 1% pentobarbital sodium at a dose of 40–50 mg/kg. After anesthesia, the abdominal cavity was opened to expose the left kidney and ureter. The distal ureter was ligated at two points near the lower pole of the kidney, and the segment between ligations was excised. The abdominal cavity was irrigated with physiological saline before closure. Mice were allowed to recover under warm conditions before being returned to their cages. Sham-operated mice underwent the same surgical procedure except for ureteral ligation and transection. Blood and kidney tissue samples were collected at 3, 7and 14 days (d) post-operation. Serum creatinine (SCR)and blood urea nitrogen (BUN) were measured using an automated biochemical analyzer.

### Histopathological examination and scoring of renal tissue

After blood collection under anesthesia, renal tissues were harvested from the model mice, fixed in 4% paraformaldehyde, embedded in paraffin, and sectioned at 2 μm thickness. Sections were stained with Periodic Acid-Schiff (PAS) for histological observation. Renal fibrosis was assessed using Sirius Red staining. For each section, ten non-overlapping fields were randomly selected and examined under a light microscope at 400× magnification. Sirius Red-positive areas, appearing red, were identified as fibrotic regions. The extent of fibrosis was semi-quantitatively scored based on the percentage of the red-stained area per field according to the following criteria: 0% (score 0; No fibrosis: Normal interstitial architecture with intact tubules and no collagen deposition visible under polarized light), 0–25% (score 1; Mild fibrosis: Focal areas of collagen deposition in the interstitium, with slight tubular separation and minimal tubular atrophy), 25–50% (score 2; Moderate fibrosis: Patchy interstitial collagen accumulation, noticeable tubular dilation and atrophy, and early disruption of parenchymal structure), 50–75% (score 3; Severe fibrosis: Extensive interstitial collagen deposition, widespread tubular atrophy/loss, and significant architectural distortion), and > 75% (score 4; Extreme fibrosis: Diffuse and dense collagen replacement of the interstitium, marked loss of tubules, and advanced scarring resembling end‑stage kidney pathology)^15^.

### Western blot analysis

Renal tissues or cells were lysed using RIPA lysis buffer (Beyotime, P0013B) to extract total protein. Protein concentration was determined using a BCA assay kit (Thermo Scientific, 23225). Equal amounts of protein were separated by SDS-polyacrylamide gel electrophoresis and subsequently transferred onto NC membranes. The membranes were blocked with 10% casein for 1 h at room temperature, followed by incubation with primary antibodies at 4 °C overnight. After washing, the membranes were incubated for 2 h at room temperature with horseradish peroxidase-conjugated goat anti-rabbit or anti-mouse secondary antibodies (Beyotime, Shanghai). Antigen-antibody complexes were detected using an ultrasensitive enhanced chemiluminescence substrate (Pulilai, P1030) and visualized with a Bio-Rad Quantity One imaging system. All methods were performed in accordance with the relevant guidelines and regulations.

Protein band intensities were semi-quantified using ImageJ software (National Institutes of Health, USA). The following primary antibodies were used: α-SMA (Proteintech, 67735-1, 1:10000), vimentin (Abcam, ab92547, 1:2000), Collagen I (Proteintech, 14695-1-AP, 1:2000), p-NF-κB (p-p65 Wanlei Bio, WL02169, 1:1000), Nuclear Factor Kappa-Light-Chain-Enhancer of Activated B Cells (NF-κB) (p65 Wanlei Bio, WL01980, 1:1000), p-Inhibitor of Kappa B Alpha (p-IκBα Wanlei Bio, WL02495, 1:1000), IκBα (Wanlei Bio, WL01936, 1:1000), Interleukin(IL)-1β (Abcam, ab254360, 1:1000), and IL-6 (Abcam, ab290735, 1:1000). Gapdh (Proteintech, 60004-1, 1:50000) and β-actin (Proteintech, 20536-1, 1:10000) were used as internal controls for normalization.

### Immunofluorescence staining

Tissue sections were deparaffinized in xylene and rehydrated through a graded ethanol series, followed by rinsing in phosphate-buffered saline (PBS). Antigen retrieval was performed by microwave heating in 10 mmol/L citrate buffer (pH 6.0) for 15 min. The sections were then blocked with 1.5% goat serum for 1 h at room temperature and incubated overnight at 4 °C with the following primary antibodies: α-SMA (Abcam, ab7817, 1:1000), vimentin (Beyotime, AF0318, 1:200), and collagen IV (Abcam, ab6586, 1:500). After washing, the sections were incubated for 2 h at room temperature with species-appropriate secondary antibodies, including goat anti-mouse IgG and goat anti-rabbit IgG (Beyotime, Shanghai). Fluorescence images were acquired using a confocal laser scanning microscope (Olympus, Japan) and analyzed with ImageJ software (National Institutes of Health, USA).

### Cell culture and treatment

The harvested renal tissue was digested with type I collagenase (4 mg/mL; Sigma-Aldrich, SCR103) at 37 °C for 30 min. After centrifugation, the cell pellet was resuspended in PBS. The suspension was sequentially filtered through 70 μm and 40 μm cell strainers. Primary TECs were cultured in RPMI 1640 medium supplemented with 10% fetal bovine serum and 1% penicillin–streptomycin at 37 °C under 5% CO₂. Cells were treated with 50 ng/mL SCF (R&D Systems, 455-MC-020/CF) and 5 µmol/L SC-75741 (MedChemExpress, HY-10496) for 48 h prior to harvesting for subsequent experiments.

### Statistical analysis

All experiments were performed with at least three biological replicates, and results are presented as mean ± standard deviation. Pairwise comparisons were conducted using the t-test. Comparisons among multiple groups for a single variable were performed using one-way analysis of variance (ANOVA), while comparisons among multiple groups for two independent variables were performed using two-way ANOVA. If the overall ANOVA result was statistically significant, Tukey’s HSD test was used for post hoc pairwise comparisons. All statistical analyses and graphing were performed using GraphPad Prism 8.0 software. A *P*-value < 0.05 was considered statistically significant. Renal transcriptome data from the 14-day unilateral ureteral obstruction model were obtained from the GSE261665 and GSE198962 datasets in the Gene Expression Omnibus (GEO) database. Gene set enrichment analysis (GSEA) of the combined dataset was performed using R version 4.5.0.

## Results

### Upregulation of SCF/c-kit and fibrosis markers in renal tissues of mice after UUO

**Fig. 1:**
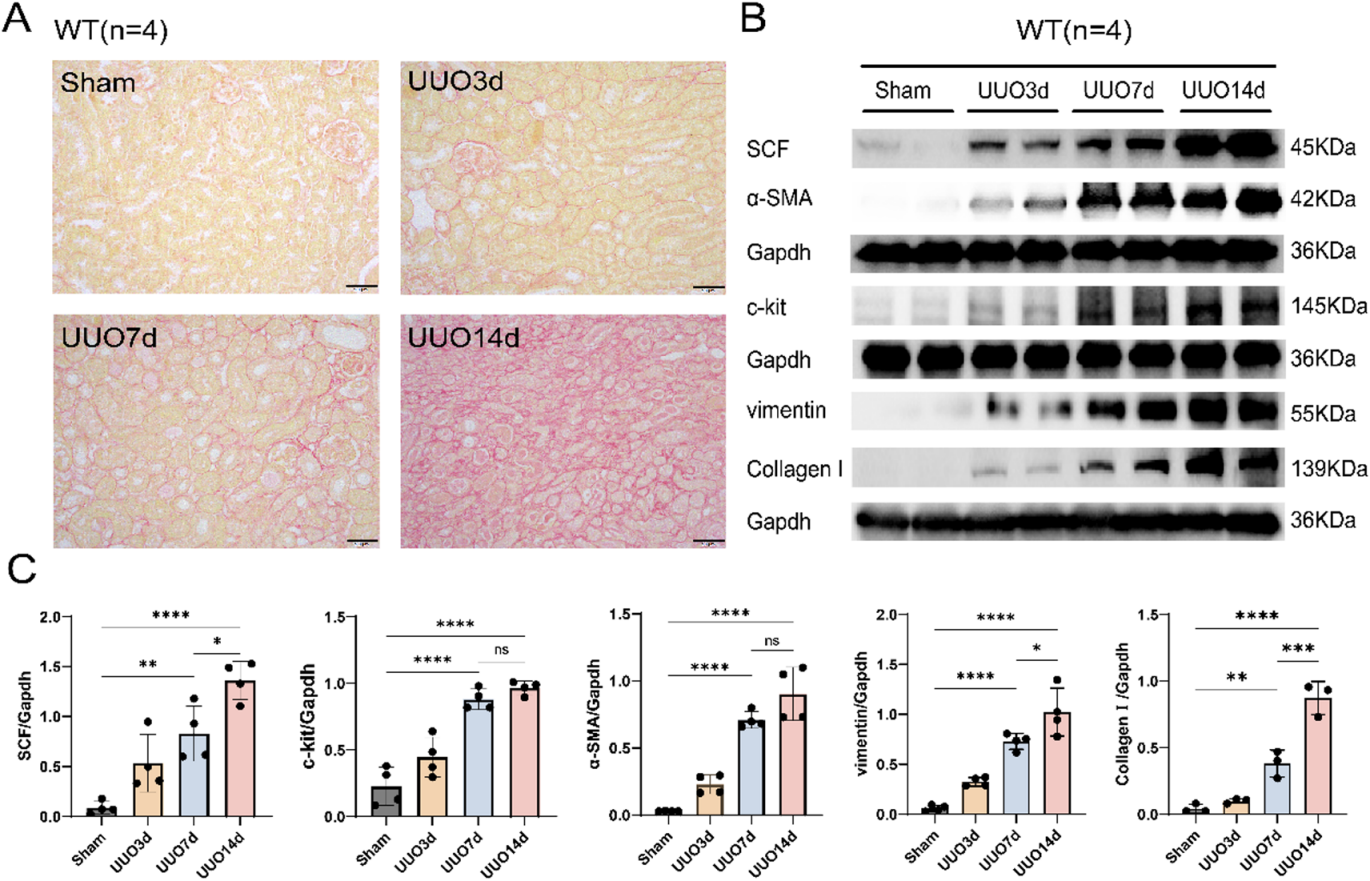
Upregulation of SCF/c-kit and Fibrosis Markers in Renal Tissues of Mice After UUO Surgery. **A** Sirius red staining revealed progressively aggravated renal interstitial fibrosis in the WT group at UUO 3, 7, and 14d (40×, *n* = 4). **B** Western blot analysis of the WT group showed that the expression of SCF and c-kit was significantly increased at UUO 7 and 14d, with a marked increase at 14d. A consistent expression trend was observed for the fibrosis-related proteins α-SMA, Vimentin, and Collagen I (*n* = 4). ** C** Quantitative analysis of the expression levels of SCF, c-kit, and fibrosis-related proteins (α-SMA, Vimentin, Collagen I), normalized to Gapdh. Data are presented as mean ± standard deviation. *: *P* < 0.05; **: *P* < 0.01; ***: *P* < 0.001; ****: *P* < 0.0001. WT: Wild Type; UUO: Unilateral Ureteral Obstruction; SCF: Stem Cell Factor; α-SMA: Alpha-Smooth Muscle Actin.

Kidney tissues were harvested at 3, 7, and 14d after UUO surgery and subjected to Sirius red staining. The deposition of red-stained collagen fibers increased progressively over time: focal and fine collagen bundles were observed at 3d; thickened and reticular collagen tracts were evident at 7d; and extensive dense collagen deposition was noted at 14d, indicating progressively aggravated renal interstitial fibrosis (Fig. 1A). 

Further analysis revealed that, compared with the Sham group, the expression levels of fibrosis-related proteins, including α-SMA, vimentin, and Collagen I, were gradually upregulated after UUO surgery, with significant increases observed at 7 and 14d. The expression of SCF and its receptor c-kit showed a similar increasing trend, consistent with the changes in fibrosis-related proteins (Fig. 1B, C). These results suggest that the SCF/c-kit signaling pathway may be involved in the fibrotic process following UUO-induced kidney injury.

### Deletion of c-kit in TECs ameliorates the early decline in renal function in UUO model mice

**Fig. 2:**
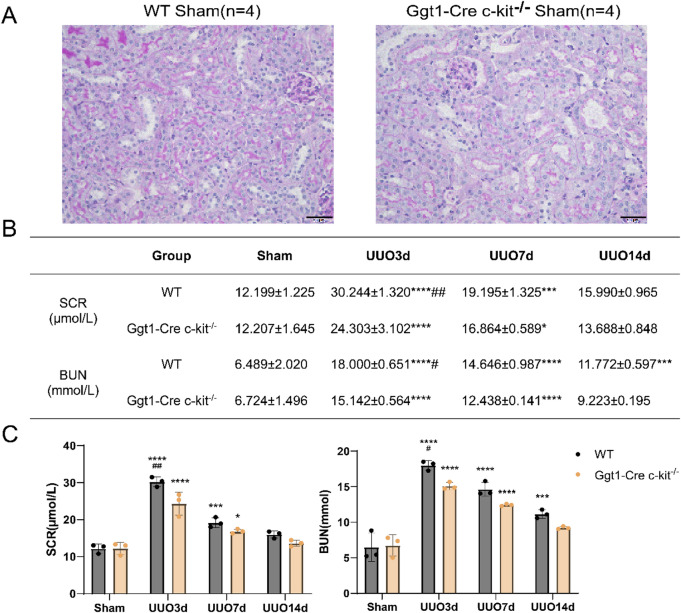
Deletion of c-kit in TECs Ameliorates the early decline in Renal Function in UUO Model Mice. ** A** PAS staining of kidney tissues from WT and Ggt1‑Cre c‑kit^−/−^ Sham mice. No significant differences were observed in PAS staining between WT and Ggt1-Cre c-kit^−/−^ Sham mice (40×, *n* = 4). ** B** Effect of c-kit gene knockout on renal function in UUO mice. Changes in SCR and BUN levels in WT and Ggt1‑Cre c‑kit−/− mice at Sham, and UUO 3, 7, and 14d(*n* = 3). ** C** Two‑way ANOVA bar graph showing SCR and BUN changes in WT and Ggt1‑Cre c‑kit−/− mice at Sham and UUO 3, 7, and 14d (*n* = 3). Data are presented as mean ± standard deviation. *: *P* < 0.05; **: *P* < 0.01; ***: *P* < 0.001; ****: *P* < 0.0001. #:*P* < 0.05; ##: *P* < 0.01. WT: Wild Type; TECs: Tubular Epithelial Cells; UUO: Unilateral Ureteral Obstruction; SCR: Serum Creatinine; BUN: Blood Urea Nitrogen; ANOVA: analysis of variance.

 PAS staining revealed no significant renal pathological damage in Ggt1-Cre c-kit^−/−^ Sham mice compared with WT Sham mice. The corticomedullary architecture remained intact, with normal glomerular size and morphology, and no evident inflammatory cell infiltration or collagen deposition in the renal interstitium (Fig. 2A). 

Regarding renal function indices, no significant differences were observed in serum creatinine and blood urea nitrogen between the WT and Ggt1-Cre c-kit^−/−^ Sham groups, indicating that genetic knock-out had no impact on renal function. After establishment of the UUO, SCR and BUN in both the WT and Ggt1-Cre c-kit^−/−^ groups were significantly elevated on 3d compared with their respective Sham groups, followed by a gradual decline thereafter. Compared with the WT group, the Ggt1-Cre c-kit^−/−^ group exhibited significantly lower levels of serum creatinine and blood urea nitrogen on UUO 3d, while no significant differences were observed on 7 and 14d. These findings suggest that the primary effect of c-kit gene knock-out is to attenuate the early and acute renal function impairment induced by UUO (Fig. 2B,C).

### Deletion of c-kit in TECs attenuates RIF in the UUO model

**Fig. 3:**
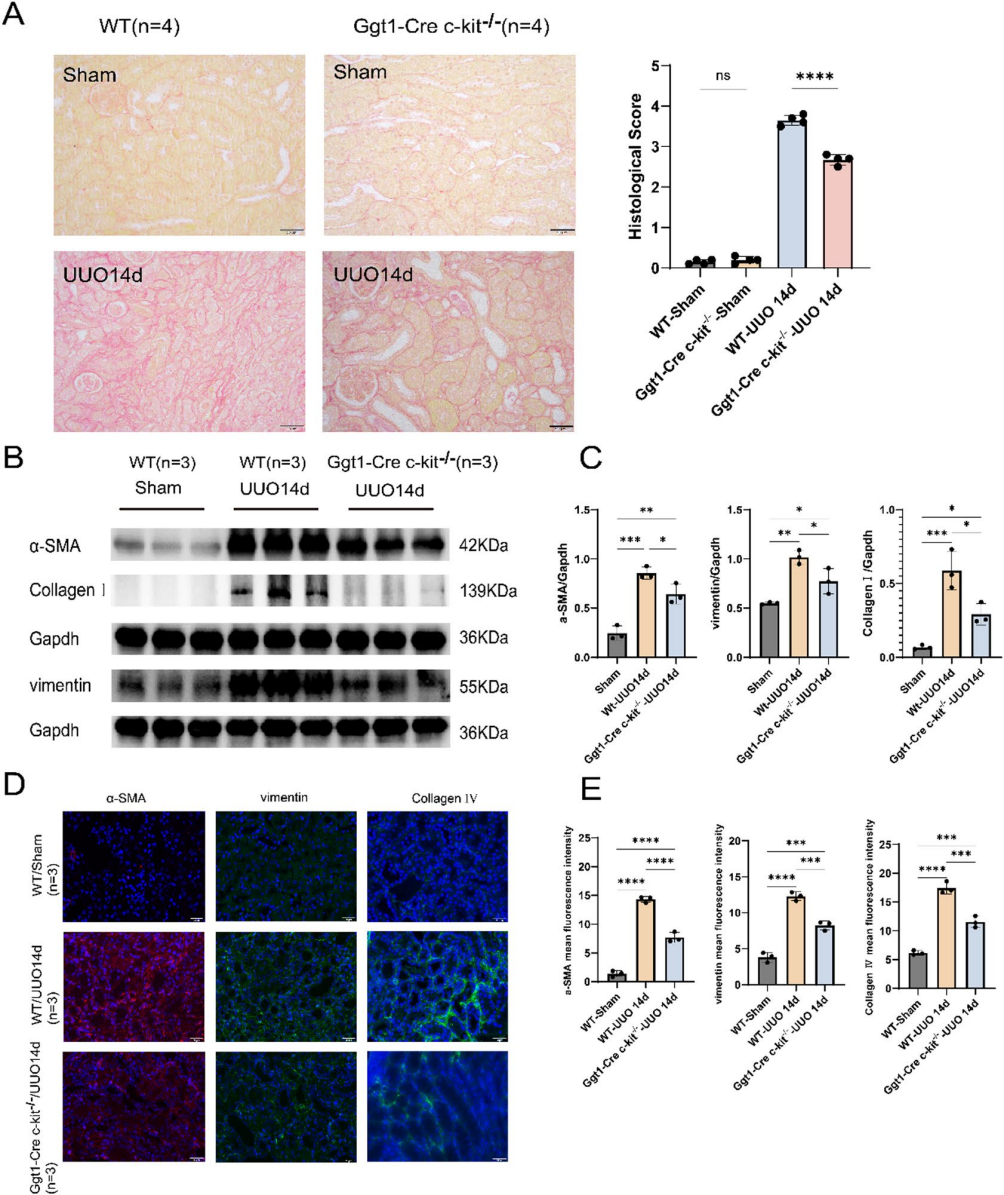
Deletion of c-kit in TECs attenuates RIF in the UUO model. **A** Sirius Red staining results and a bar graph showing semi-quantitative assessment of interstitial fibrotic lesions using NIH scoring criteria in WT and Ggt1-Cre c-kit^−/−^ subjected to UUO for 14d (40×, *n* = 4). The fibrotic area was significantly reduced in the Ggt1-Cre c-kit−/− compared to the WT at UUO14d. ** B**, **C** Western blot analysis and corresponding bar graphs showing expression levels of fibrosis-related markers (α-SMA, Vimentin, and Collagen I) in WT Sham, WT UUO 14d, and Ggt1-Cre c-kit^−/−^ UUO 14d groups (normalized to Gapdh, *n* = 3). Compared to the WT Sham, both WT UUO and Ggt1-Cre c-kit^−/−^ UUO showed significant increases in the expression of α-SMA, Vimentin, and Collagen I. At UUO 14d, the WT exhibited markedly higher expression of these markers than the Ggt1-Cre c-kit^−/−^. ** D**, **E** Immunofluorescence staining of fibrosis-related markers (α-SMA, Vimentin, and Collagen IV) and bar graphs showing mean fluorescence intensity in WT Sham, WT UUO 14d, and Ggt1-Cre c-kit^−/−^ UUO 14d (*n* = 3). The expression trends were consistent with those observed in Western blot analysis. Data are presented as mean ± standard deviation. *: *P* < 0.05; **: *P* < 0.01; ***: *P* < 0.001; ****: *P* < 0.0001. WT: Wild Type; TECs: Tubular Epithelial Cells; UUO: Unilateral Ureteral Obstruction; α-SMA: Alpha-Smooth Muscle Actin; RIF: Renal Interstitial Fibrosis.

In WT mice at UUO 14d, renal tissue exhibited significant pathological injury, including tubular epithelial cell swelling, necrosis and detachment, cast formation, interstitial expansion, and extensive fibrosis. In comparison, Ggt1-Cre c-kit^−/−^ mice showed markedly attenuated renal damage and reduced interstitial fibrosis area under the same conditions (Fig. 3A).

Consistent with the histological findings, the expression levels of fibrosis-related proteins (α-SMA, vimentin, and Collagen I) were significantly higher in WT mice at UUO 14d. Although these proteins were also elevated in Ggt1-Cre c-kit^−/−^ mice, their expression levels were substantially lower than those in WT mice (Fig. 3B,C).

Immunofluorescence analysis of kidney sections at UUO 14d further supported these results. The mean fluorescence intensities of fibrosis-related markers (α-SMA, vimentin, and Collagen IV) were higher in both WT and Ggt1-Cre c-kit^−/−^ UUO groups compared to their respective sham controls. However, the Ggt1-Cre c-kit^−/−^ group exhibited significantly lower fluorescence intensity than the WT UUO group (Fig. 3D,E). These data collectively indicate that conditional deletion of c-kit in TECs attenuates UUO-induced RIF.

### SCF/c-kit exacerbates RIF in UUO mice via activation of the NF-κB pathway

**Fig. 4:**
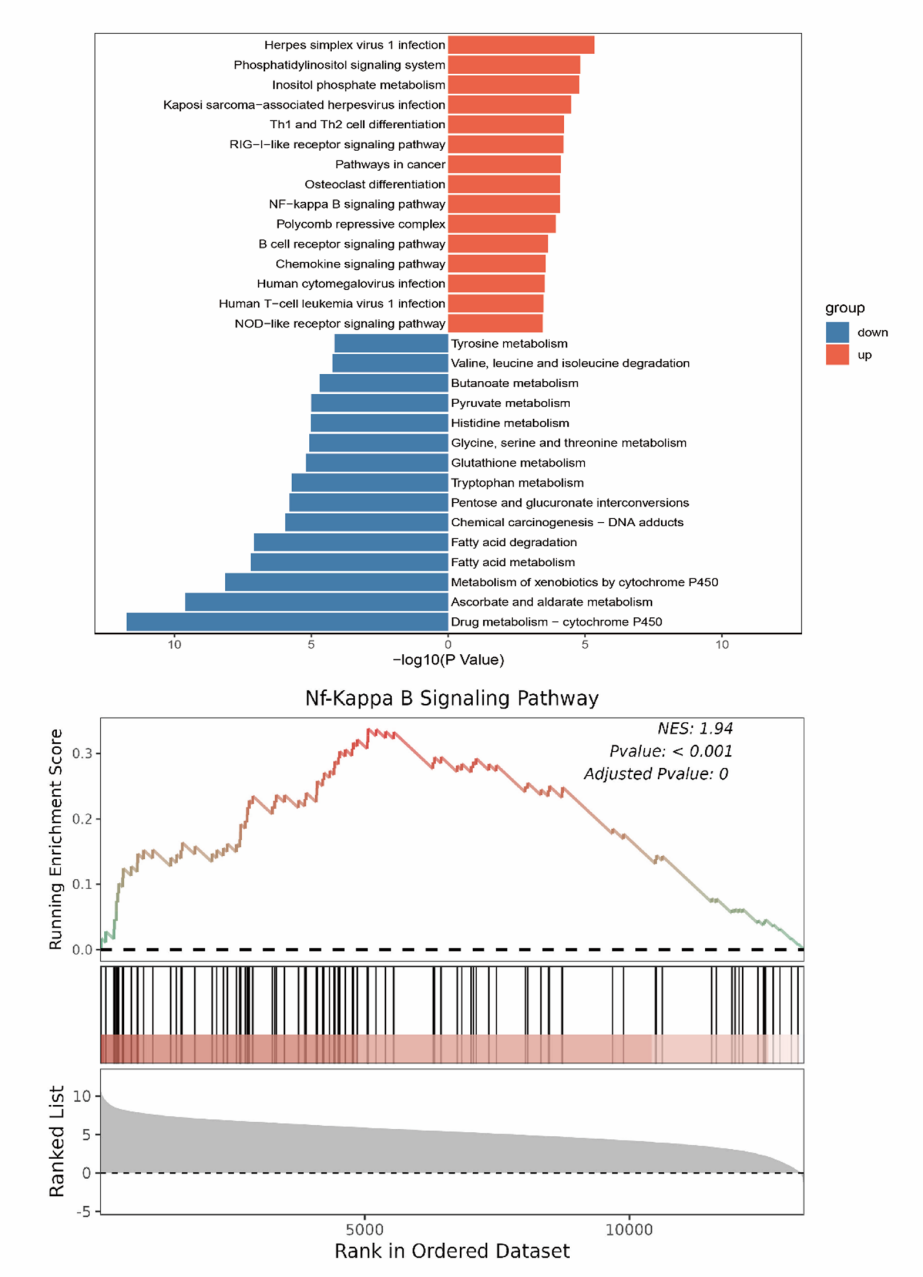
GSEA revealed a positive correlation between the NF-κB signaling pathway and c-kit expression during UUO-induced fibrosis. GSEA: Gene Set Enrichment Analysis; UUO: Unilateral Ureteral Obstruction; NF-κB: Nuclear Factor Kappa-Light-Chain-Enhancer of Activated B Cells.

**Fig. 5:**
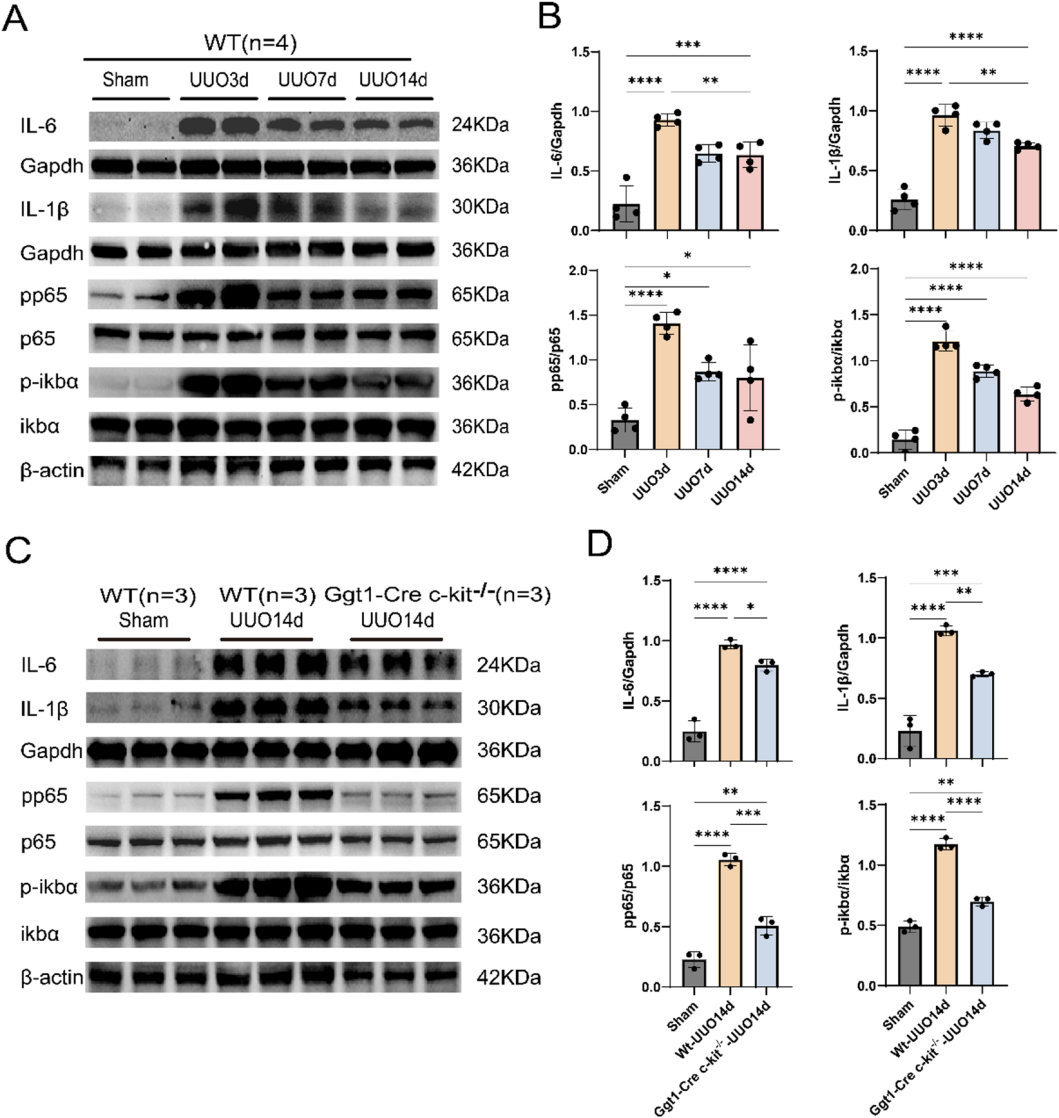
SCF/c-kit aggravates RIF in UUO mice via activation of the NF-κB pathway. **A** Western blot analysis of WT showed that UUO-induced fibrosis was accompanied by increased expression of the inflammatory cytokines IL-6 and IL-1β, as well as activation of the NF-κB signaling pathway, as indicated by elevated pp65/p65 and p-IκBα/IκBα ratios. These changes were most pronounced at 3d (*n* = 4). **C** Western blot analysis demonstrated that, compared with the WT Sham, both WT and Ggt1-Cre c-kit^−/−^ subjected to UUO 14d exhibited increased expression of IL-6 and IL-1β and activation of the NF-κB pathway (elevated pp65/p65 and p-IκBα/IκBα ratios). At UUO 14d, these increases were significantly more pronounced in WT than in Ggt1-Cre c-kit^−/−^. (*n* = 3). ** B**, **D** Quantitative analysis of the expression levels of IL-6, IL-1β, and NF-κB pathway-related proteins (pp65/p65, p-IκBα/IκBα), normalized to Gapdh and β-actin. Data are presented as mean ± standard deviation. *P < 0.05; **P < 0.01; ***P < 0.001; ****P < 0.0001. WT: Wild Type; TECs: Tubular Epithelial Cells; UUO: Unilateral Ureteral Obstruction; RIF: Renal Interstitial Fibrosis; IκBα: inhibitor of kappa B alpha; NF-κB: Nuclear Factor Kappa-Light-Chain-Enhancer of Activated B Cells; IL: Interleukin.

GSEA analysis of renal transcriptomic data from public databases (GSE261665 and GSE198962) at UUO14d revealed a positive correlation between the NF-κB signaling pathway and c-kit expression, suggesting that c-kit may promote UUO-induced renal inflammation and subsequent interstitial fibrosis through activation of NF-κB (Fig. 4). Following UUO surgery, the expression of inflammatory cytokines IL-6 and IL-1β was upregulated, peaking at 3d and gradually declining thereafter, though levels remained significantly elevated at 14d. The phosphorylation ratios of p-NF-κB/t-NF-κB (p-p65/p65) and p-IκBα/IκBα showed trends consistent with IL-6 and IL-1β expression (Fig. 5A, B). Furthermore, at UUO 14d, both WT mice and Ggt1-Cre c-kit^−/−^ mice exhibited upregulation of the inflammatory cytokines IL-6 and IL-1β, as well as increased ratios of pp65/p65 and p-IκBα/IκBα. However, the elevation of these ratios was significantly lower in Ggt1-Cre c-kit^−/−^ mice compared to the WT mice (Fig. 5C,D). These results indicate that SCF/c-kit signaling likely contributes to RIF in the UUO model by activating the NF-κB pathway in TECs.

### In vitro experiments confirm that SCF/c-Kit promotes TECs fibrosis via NF-κB pathway activation

**Fig. 6:**
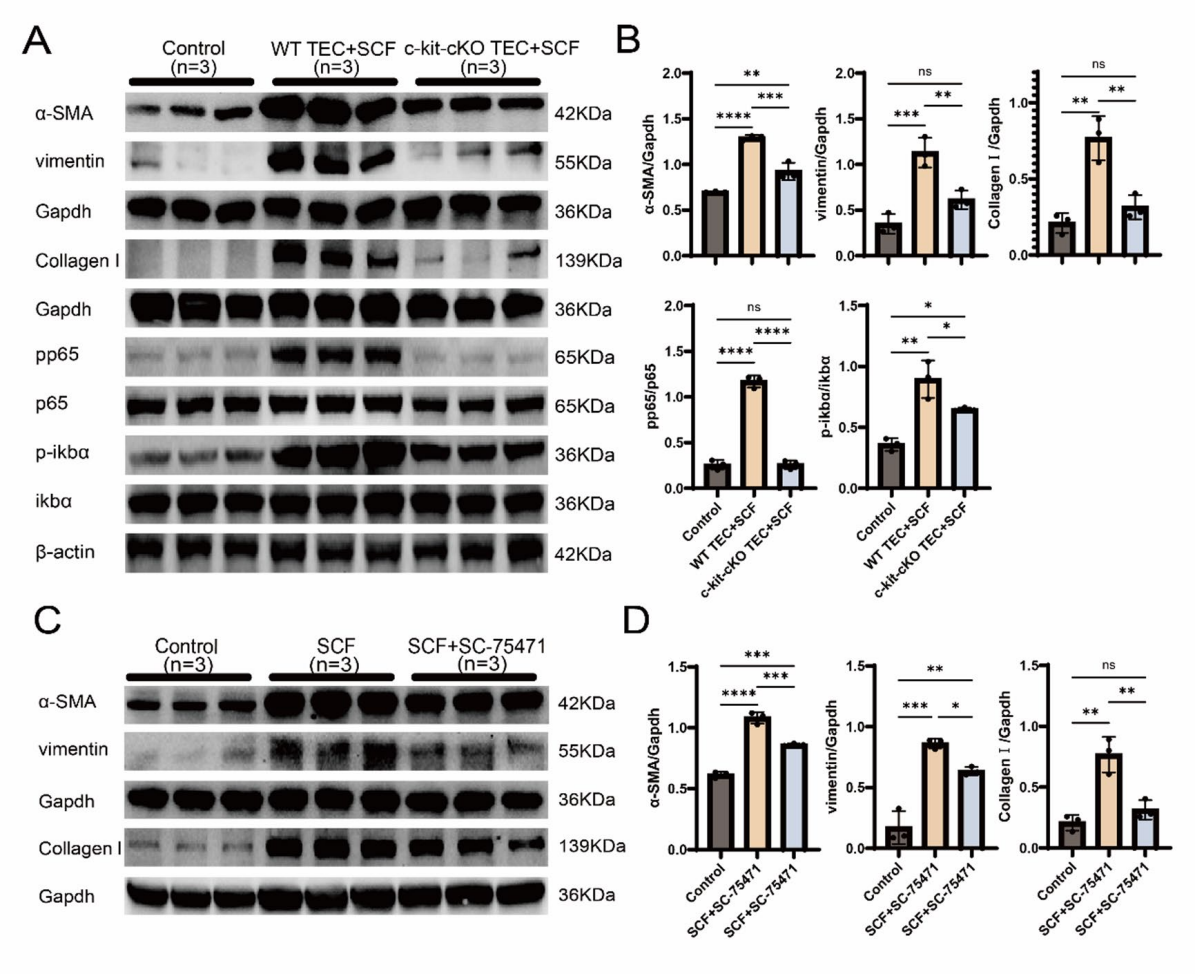
In Vitro Experiments Confirm That SCF/c-Kit Promotes TECs Fibrosis via NF-κB Pathway Activation. **A** Western blot analysis showed that after SCF stimulation, the expression of α-SMA, vimentin, and collagen I was significantly increased in the WT compared to the control and c-kit cKO. Except for α-SMA, no statistically significant differences were observed in other markers between the control and c-kit cKO. The ratios of pp65/p65 and p-IκBα/IκBα in the WT were significantly higher than those in the other two groups, while no significant difference in pp65/p65 was detected between the c-kit cKO and control. (*n* = 3). C Western blot results indicated that the expression of fibrosis markers (α-SMA, vimentin, collagen I) was higher in both the SCF and SCF + SC-75741 groups compared to the control, with the most significant upregulation observed in the SCF group. (*n* = 3). **B**, **D** Quantitative bar charts of fibrosis-related proteins (α-SMA, vimentin, collagen I) and NF-κB pathway proteins (pp65/p65, p-IκBα/IκBα) normalized to Gapdh and β-actin. Data are presented as mean ± standard deviation. *P < 0.05, **P < 0.01, ***P < 0.001, ****P < 0.0001. WT: Wild Type; SCF: Stem Cell Factor; TECs: Tubular Epithelial Cells; UUO: Unilateral Ureteral Obstruction; α-SMA: Alpha-Smooth Muscle Actin; IκBα: inhibitor of kappa B alpha; NF-κB: Nuclear Factor Kappa-Light-Chain-Enhancer of Activated B Cell.

Primary TECs isolated from WT and Ggt1-Cre c-kit^−/−^ mice were stimulated with SCF. WT cells exhibited an elongated spindle morphology, widened intercellular spaces, and a transition toward a fibrotic phenotype. In contrast, c-kit cKO TECs showed no noticeable changes compared to the control group (Supplementary Fig. 3). Western blot analysis revealed significantly higher protein expression of α-SMA, vimentin, and Collagen I in WT TECs after SCF treatment compared to both control and c-kit cKO TECs. No statistically significant differences were observed in vimentin and Collagen I expression between control and c-kit cKO TECs, although α-SMA was higher (Fig. 6A,B). These results suggest that the SCF/c-kit axis promotes a fibrotic phenotypic transition in TECs. The phosphorylation ratios of p-p65/p65 and p-IκBα/IκBα were markedly elevated in WT TECs compared to control and c-kit cKO groups. While the p-p65/p65 ratio in c-kit cKO TECs did not differ significantly from the control, both phosphorylation ratios were significantly suppressed compared to WT TECs (Fig. 6A,B). To further investigate the role of NF-κB signaling, WT TECs were co-treated with SCF and the NF-κB-specific inhibitor SC-75741(Supplementary Fig. 4). SC-75741 significantly attenuated the SCF-induced upregulation of α-SMA, vimentin, and Collagen I (Fig. 6C,D). These findings demonstrate that SCF/c-kit-induced fibrotic transformation in TECs is dependent on NF-κB pathway activation.

## Discussion

In this study, a renal tubular epithelial cell-specific c-kit gene knockout mouse model was constructed using the Cre-loxP system. Combined with the classic UUO fibrosis model and in vivo/in vitro experiments, the mechanism by which the SCF/c-kit axis promotes renal fibrosis in the UUO model was elucidated for the first time: SCF/c-kit can directly induce a fibrotic phenotype in renal tubular epithelial cells. Inhibition of c-kit expression in TECs alleviated the early and severe renal function impairment induced by UUO and reduced RIF by suppressing the abnormal activation of the NF-κB pathway. These findings provide a potential therapeutic target for CKD.

The SCF/c-kit signaling pathway is involved in the fibrosis and remodeling processes of multiple organs, including the lung, skin, liver, and kidney^8,16,17^. In renal diseases, elevated levels of SCF have been observed in IgA nephropathy, membranous nephropathy, crescentic glomerulonephritis, hypertensive nephropathy, and diabetic nephropathy^11,18,19^. Previous studies have primarily focused on the critical roles of SCF/c-kit and mast cells in renal fibrosis^12,20^. For instance, in crescentic glomerulonephritis, the number of interstitial mast cells is significantly increased and co-localizes with SCF, serving as an independent predictor of renal interstitial fibrosis^10^. In this study, by conditionally deleting c-kit in renal tubular epithelial cells, the confounding influence of mast cells was excluded. The specific deletion of c-kit in renal tubular epithelial cells alone still resulted in a significant anti-fibrotic effect, suggesting that renal tubular epithelial cells themselves may be the core responsive unit to SCF signaling.

In the process of renal fibrosis, transforming growth factor-beta (TGF-β) is recognized as the most potent pro-fibrotic factor. The TGF-β/Smad pathway directly promotes the synthesis of the extracellular matrix and drives renal fibrosis by facilitating ligand binding to its receptor, phosphorylation of Smad proteins, and subsequent nuclear translocation to regulate the expression of fibrosis-related genes. This study focuses on elucidating the SCF/c-kit axis, which, through the activation of receptor tyrosine kinase activity, serves as a significant upstream initiating signal, potentially by recruiting and activating multiple downstream pathways. For instance, in hyperuricemic nephropathy, elevated uric acid induces the expression of SCF and the c-kit protein in mast cells. These mast cells subsequently release Ang II, which triggers the TGF-β1/Smad2/3 pathway, thereby driving renal fibrosis^9^. This suggests that the SCF/c-kit axis may function as an important pro-fibrotic pathway independent of TGF-β/Smad. Targeting the SCF/c-kit axis could potentially be more effective in attenuating renal fibrosis than directly blocking TGF-β, which possesses broad physiological functions.

Furthermore, our previous study demonstrated that knocking out the c-kit gene in mice alleviated the expression of inflammatory factors such as TGF-β1 and NF-κB, suggesting that the c-kit gene may also amplify inflammatory responses by activating the NF-κB pathway, thereby promoting the progression of renal interstitial fibrosis^15^. In this study, GSEA of public transcriptomic data further revealed that high expression of SCF/c-kit promoted the phosphorylation of key NF-κB pathway proteins p65 and IκBα, which was consistent with the expression trends of inflammatory cytokines IL-6 and IL-1β. This indicates the involvement of SCF/c-kit in NF-κB activation during renal interstitial fibrosis following UUO. Ex vivo experiments further confirmed that SCF stimulation induced fibrotic phenotypic transformation in WT TECs, manifested as altered cell morphology and upregulation of fibrotic markers, accompanied by NF-κB pathway activation. In contrast, the NF-κB inhibitor SC-75741 reversed the SCF/c-kit-induced fibrotic phenotypic transition and collagen deposition in renal tubular epithelial cells. These findings provide a profound theoretical basis for targeting the SCF/c-kit signaling pathway to reverse renal fibrosis.

NF-κB, a key transcription factor, plays a central regulatory role in inflammation, cell death, survival, proliferation, and differentiation^21,22,23^. It serves as a critical hub in the initiation and amplification of renal interstitial fibrosis—a common pathological endpoint in almost all CKD progressing to end-stage renal disease. Activation of the NF-κB pathway in both parenchymal renal cells and immune cells promotes the release of inflammatory cytokines, contributing to glomerulosclerosis and tubulointerstitial fibrosis^24^. In the context of kidney injury, multiple stimuli activate the IKK complex, leading to phosphorylation and degradation of IκB proteins. This process releases NF-κB—primarily the p50/p65 heterodimer—allowing p65 to translocate into the nucleus, where it initiates the transcription of downstream target genes. This cascade recruit inflammatory cells, promotes myofibroblast activation, and induces ECM deposition, collectively driving renal fibrosis^25^. Consequently, targeting the NF-κB signaling pathway holds significant therapeutic potential for the treatment of kidney diseases^26^.

In addition, research on SCF/c-kit has predominantly focused on the field of oncology. Activating mutations or amplification/overexpression of c-kit can promote the development and progression of various human malignancies by constitutively activating multiple downstream signaling pathways, thereby enabling unlimited cell proliferation and survival. c-kit inhibitors (such as imatinib) have already been applied in clinical oncology^27,28^. This study reveals that knocking out c-kit in TECs can significantly alleviate the degree of renal fibrosis. It is therefore worthwhile to explore the anti-fibrotic effects and safety of c-kit inhibitors in renal fibrosis models in the future.

This study also has several limitations. Although the unilateral UUO model can induce renal fibrosis and simulate the progression of CKD, the changes in systemic blood urea nitrogen (BUN) and SCR levels result from the combined effects of the loss of function in the obstructed kidney and compensatory hyperfiltration in the contralateral kidney^29^. Therefore, the impact of inhibiting c-kit expression in TECs on renal function observed in this study requires further validation. At the mechanistic level, while this study established a causal relationship between c-kit activation and NF-κB pathway activation, the specific intermediate signaling molecules involved—such as the generation of reactive oxygen species (ROS) and alterations in Ca²⁺ flux—and whether and how they mediate c-kit–induced NF-κB activation in the present model remain to be fully elucidated in future investigations.

## Conclusion

In summary, this study demonstrates for the first time that the SCF/c-kit–NF-κB axis in TECs is a key driver of renal interstitial fibrosis induced by UUO. Targeted inhibition of c-kit in TECs not only suppresses the inflammatory response but also alleviates RIF and improves early renal function. Its significant role in delaying the progression of CKD may provide a novel therapeutic target and research direction for the treatment of CKD.

## Supplementary Information

Below is the link to the electronic supplementary material.

Supplementary Material 1

Supplementary Material 2

Supplementary Material 3

Supplementary Material 4

Supplementary Material 5

## Data Availability

The datasets analyzed in the current study are available in the NCBI GEO repository under accession numbers GSE261665 and GSE198962.

## Abbreviations

CKD: Chronic kidney disease
RIF: Renal interstitial fibrosis
SCF: Stem cell factor
TECs: Tubular epithelial cells
UUO: Unilateral ureteral obstruction
WT: Wild type
SCR: Serum creatinine
BUN: Blood urea nitrogen
PAS: Periodic acid–schiff
GSEA: Gene set enrichment analysis
ECM: Extracellular matrix
EMT: Epithelial-mesenchymal transition
PBS: phosphate-buffered saline
α-SMA: Alpha-smooth muscle actin
IκBα: inhibitor of kappa B alpha
NF-κB: Nuclear factor kappa-light-chain-enhancer of activated B cells
IL: Interleukin
ANOVA: One-way analysis of variance
GEO: Gene expression omnibus
TGF-β: Transforming growth factor-beta
ROS: Oxygen species
