# Supplementary material for: Targeted deletion of c-kit in TECs attenuates UUO-induced renal fibrosis through NF-κB pathway inhibition
Source: Sci Rep. 2026 Mar 12;16:13227. doi: 10.1038/s41598-026-42540-w (PMC13103321; doi:10.1038/s41598-026-42540-w)

Figure1 SCF original image


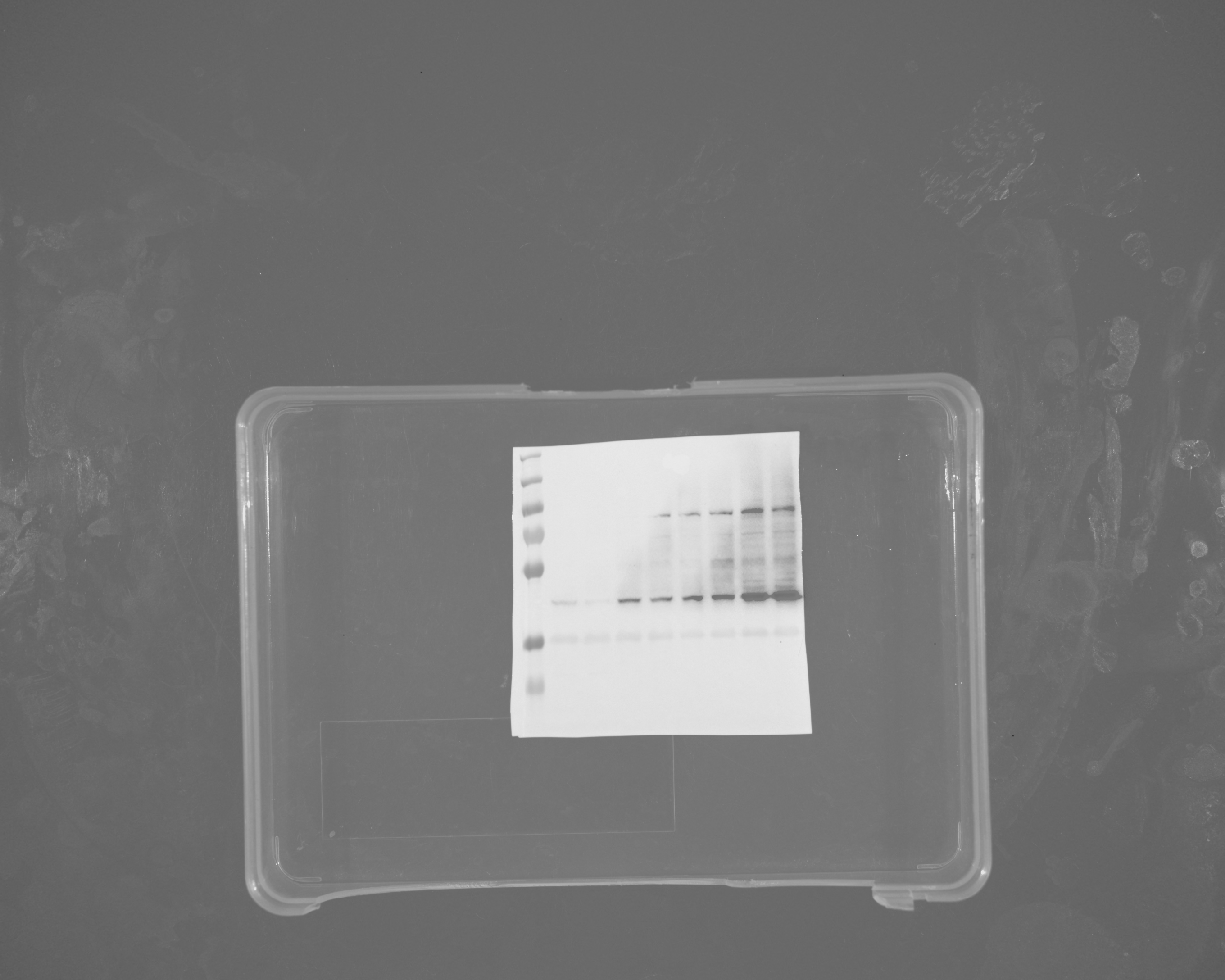


Figure1 SCF-Gapdh original image


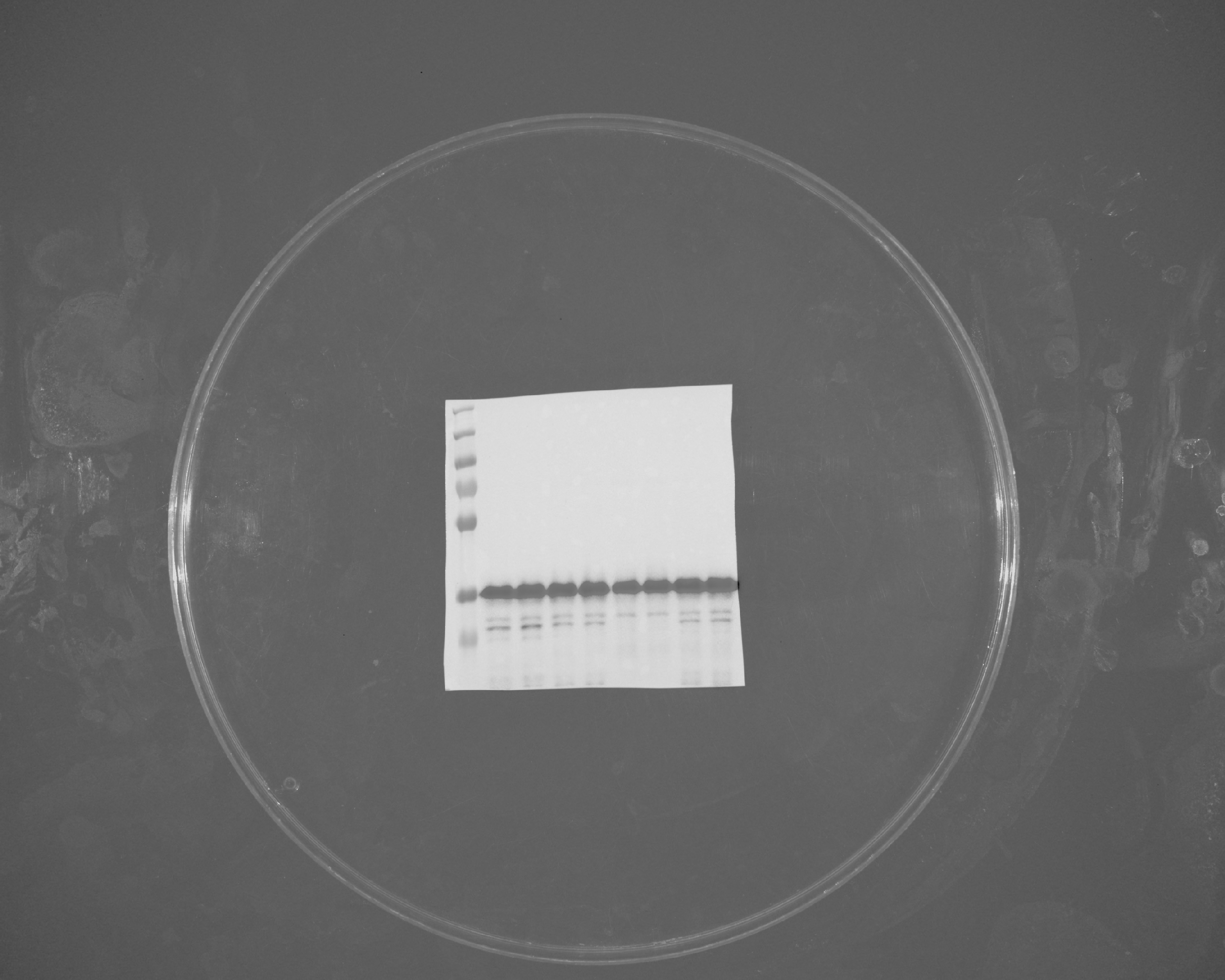


Figure1 c-kit original image


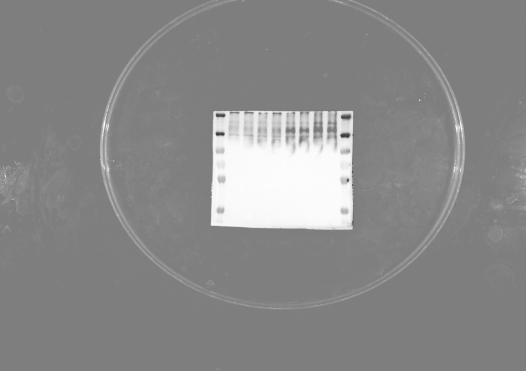


Figure1 c-kit-Gapdh original image


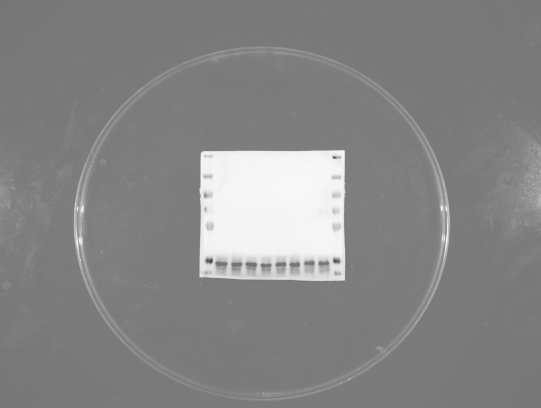


Figure1 α-SMA original image


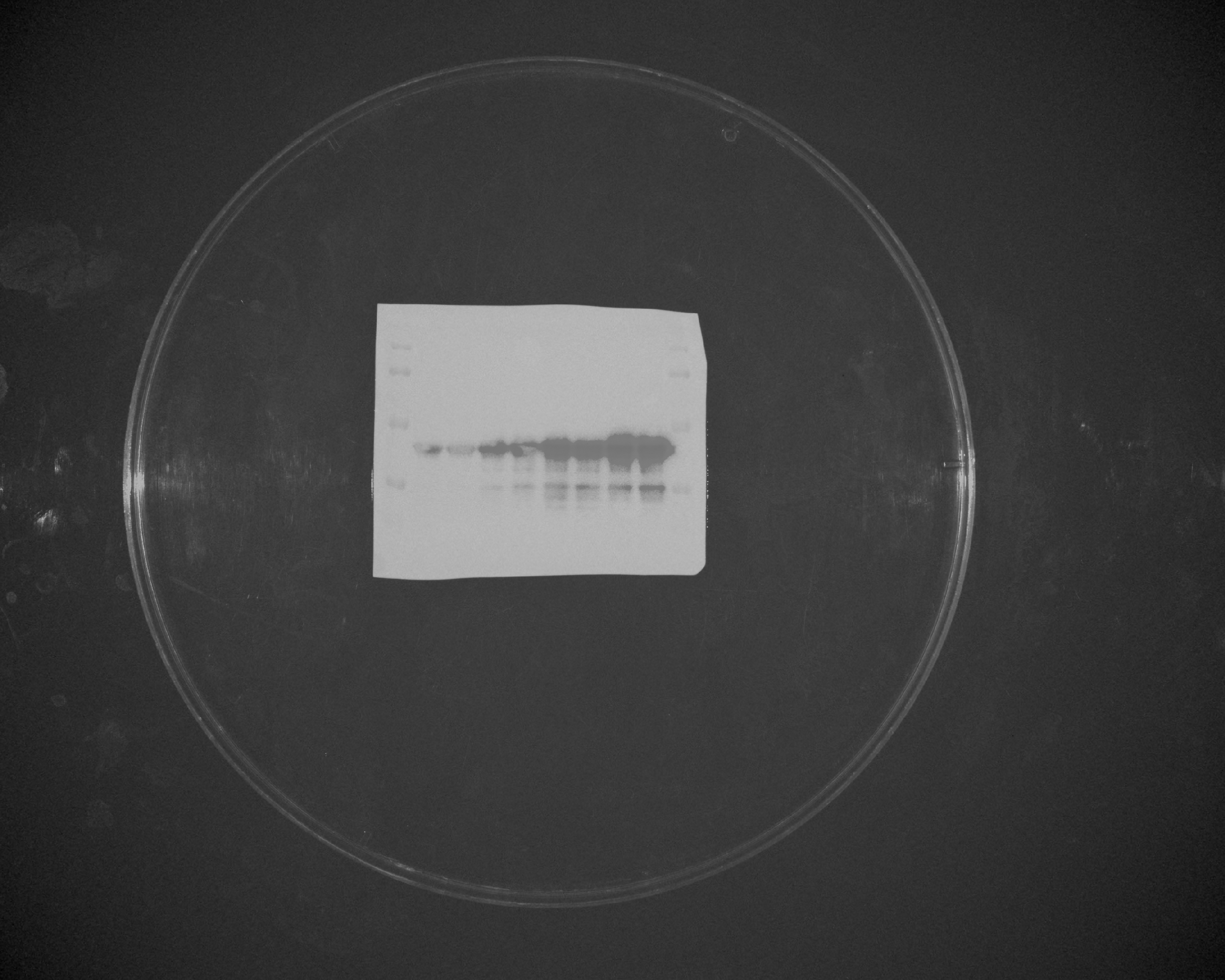


Figure1 Vimentin original image


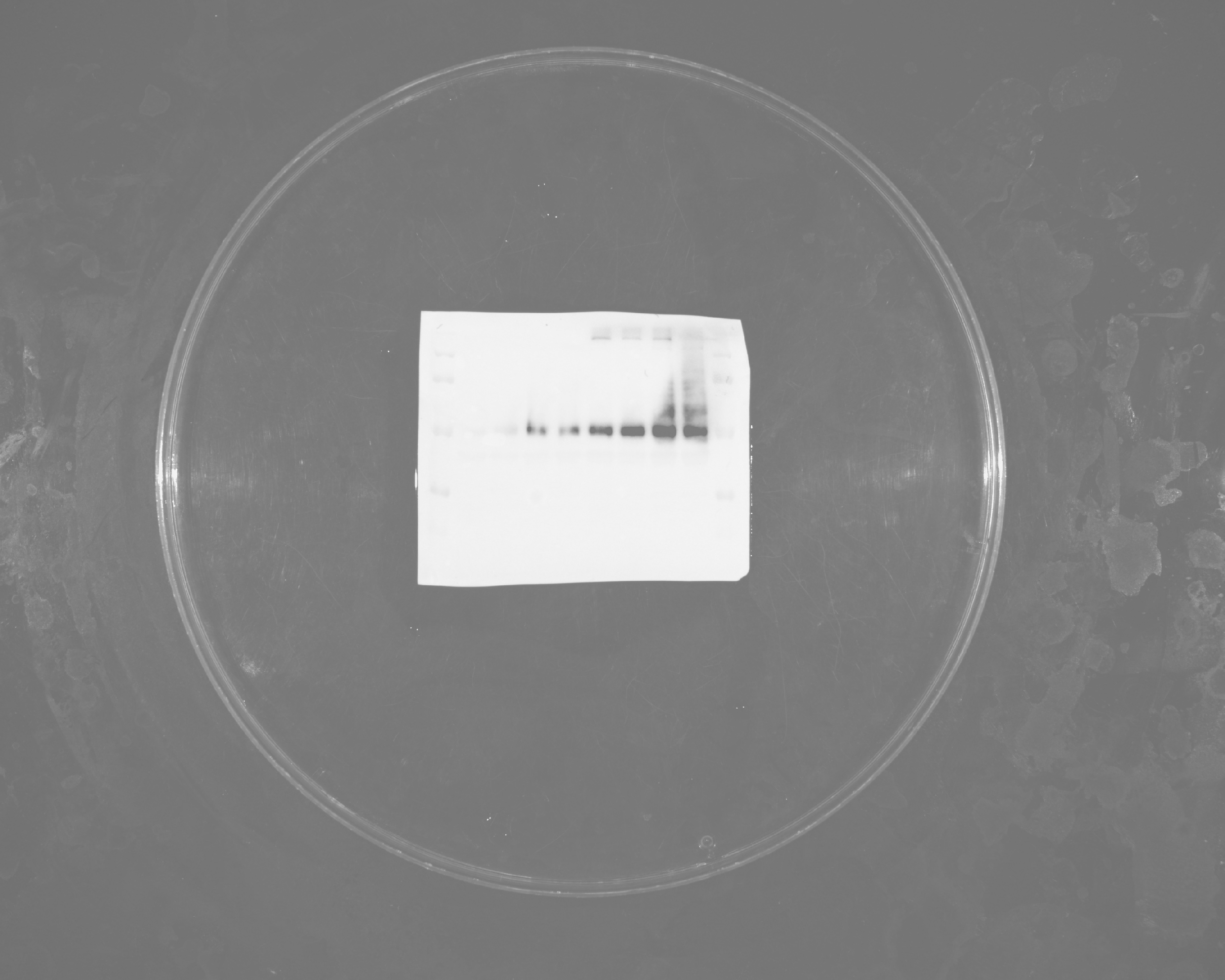


Figure1 CollagenⅠoriginal image


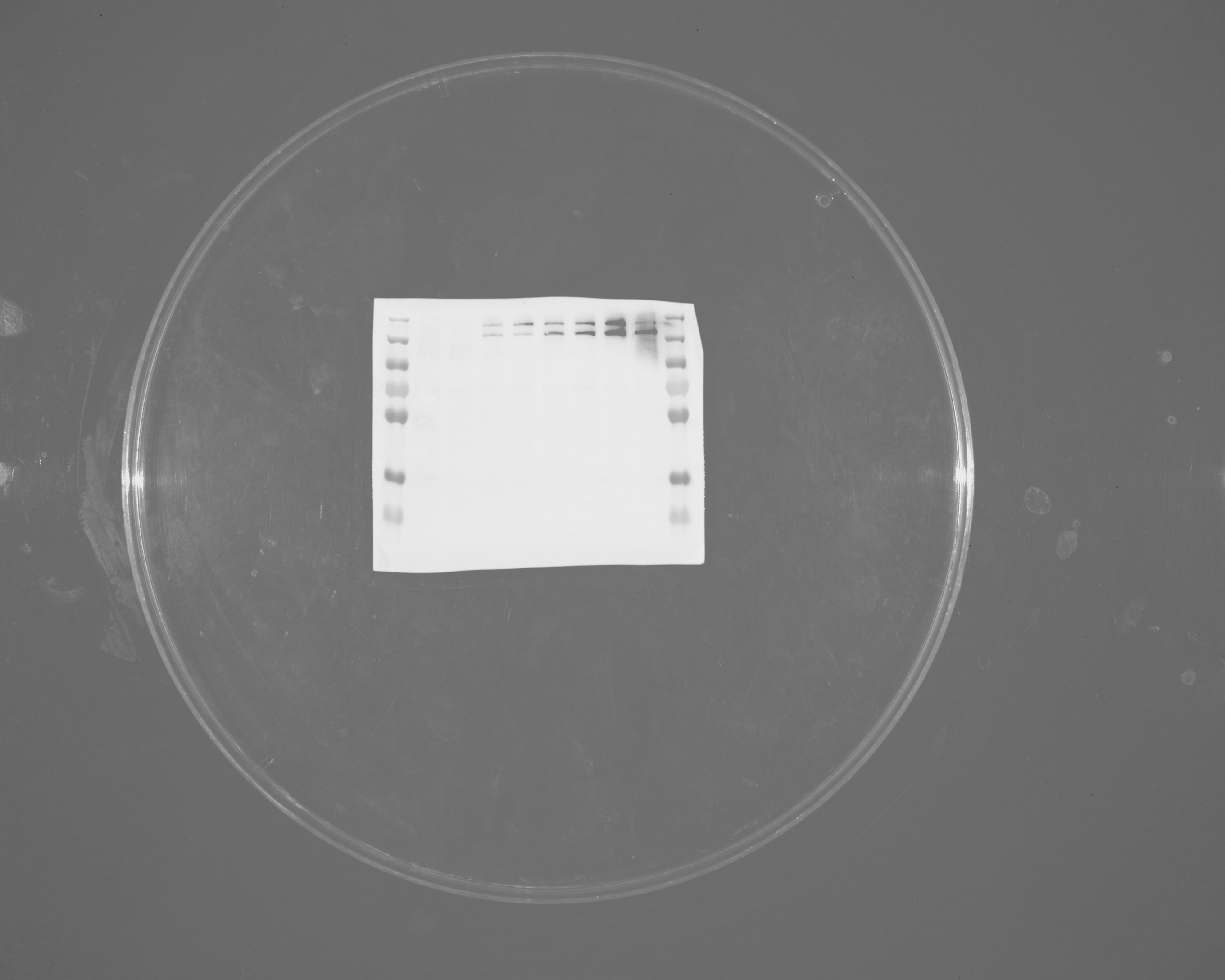


Figure1α-SMA,Vimentin,CollagenⅠ-Gapdh original image


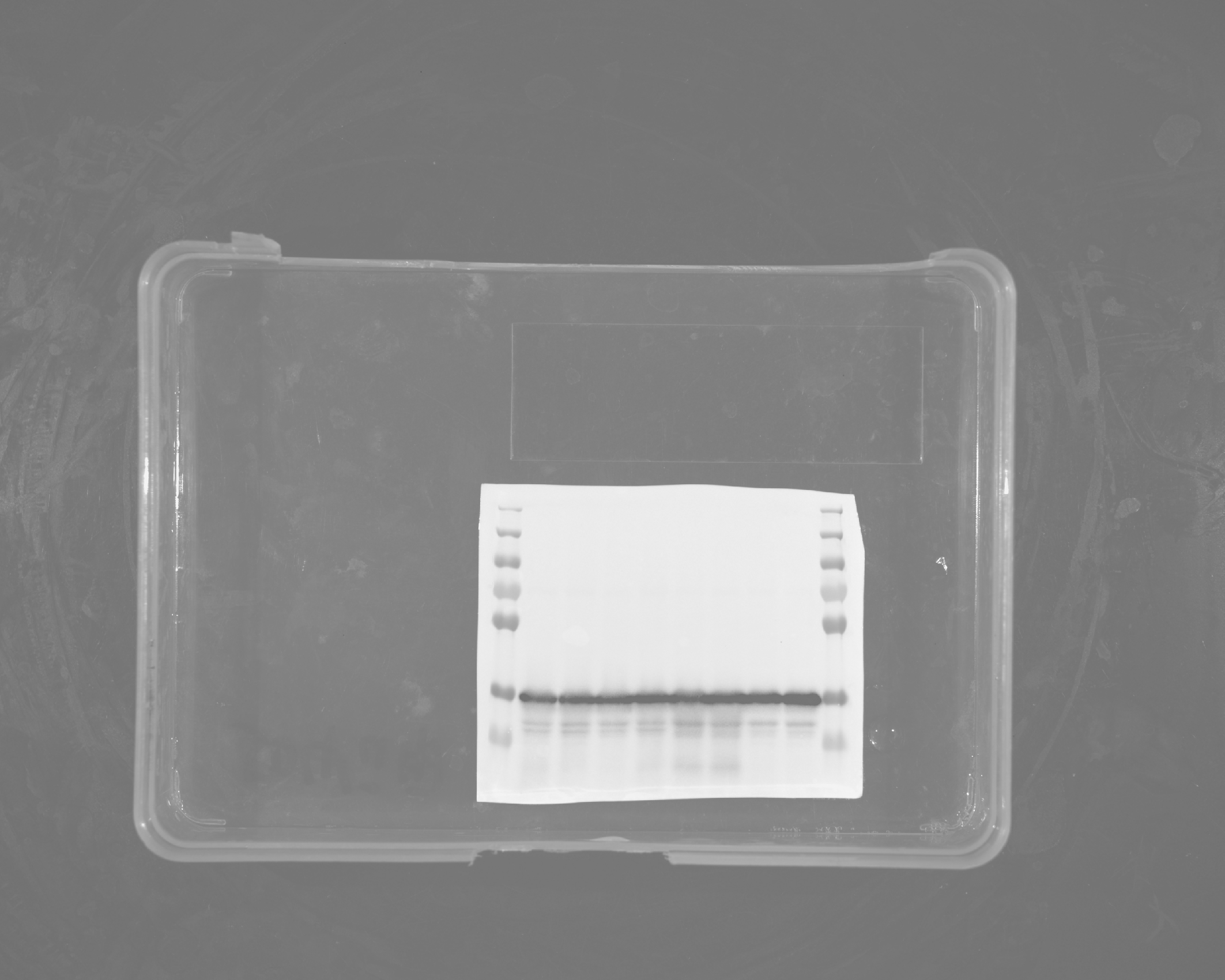


Figure3 α-SMA original image


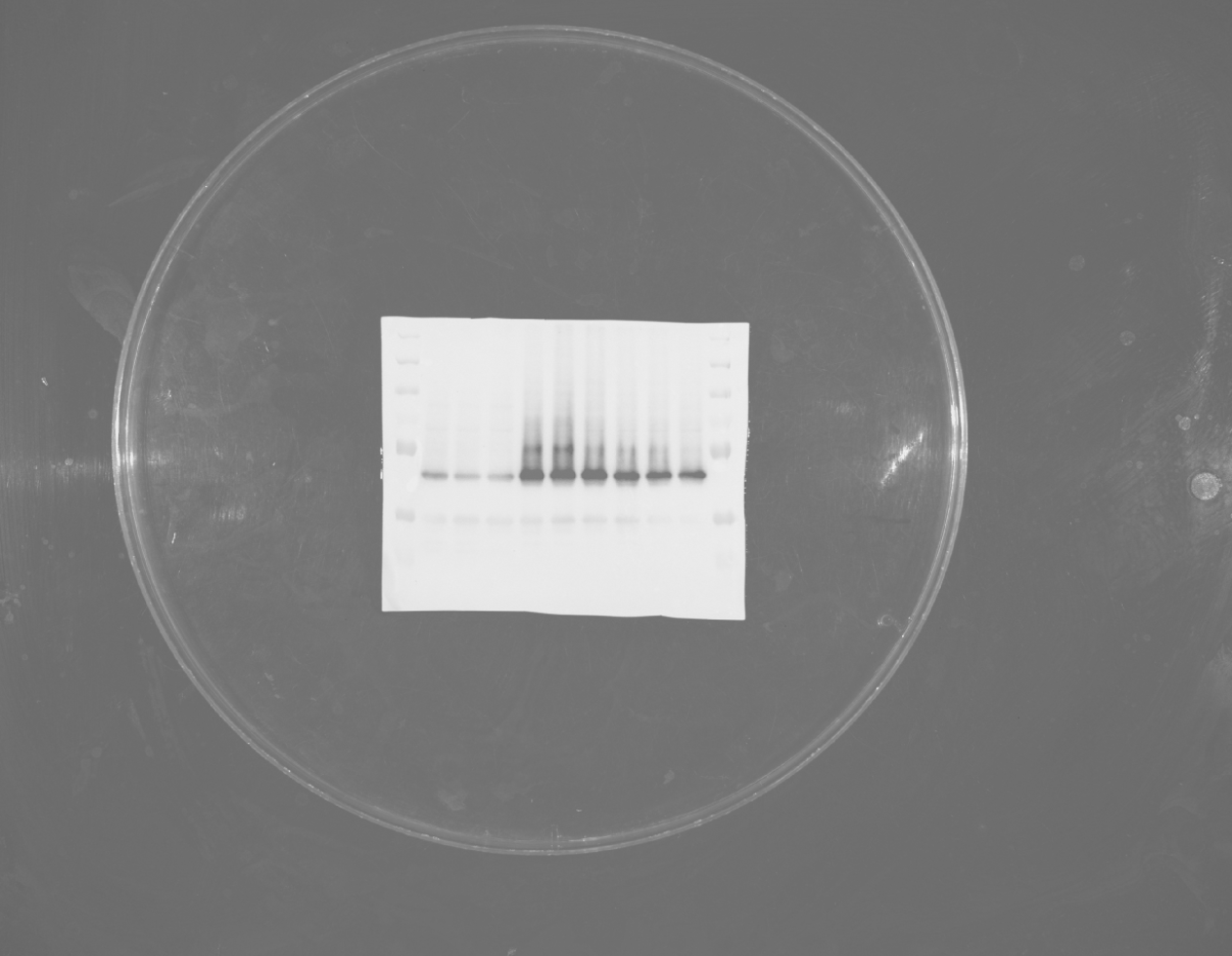


Figure3 Vimentin original image


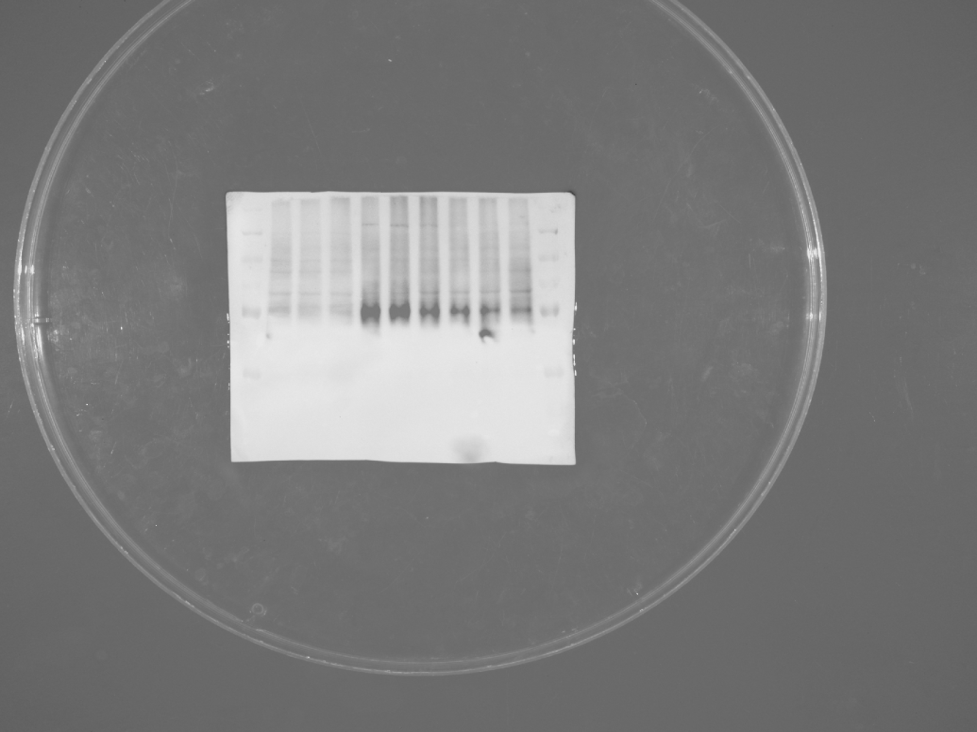


Figure3 CollagenⅠoriginal image


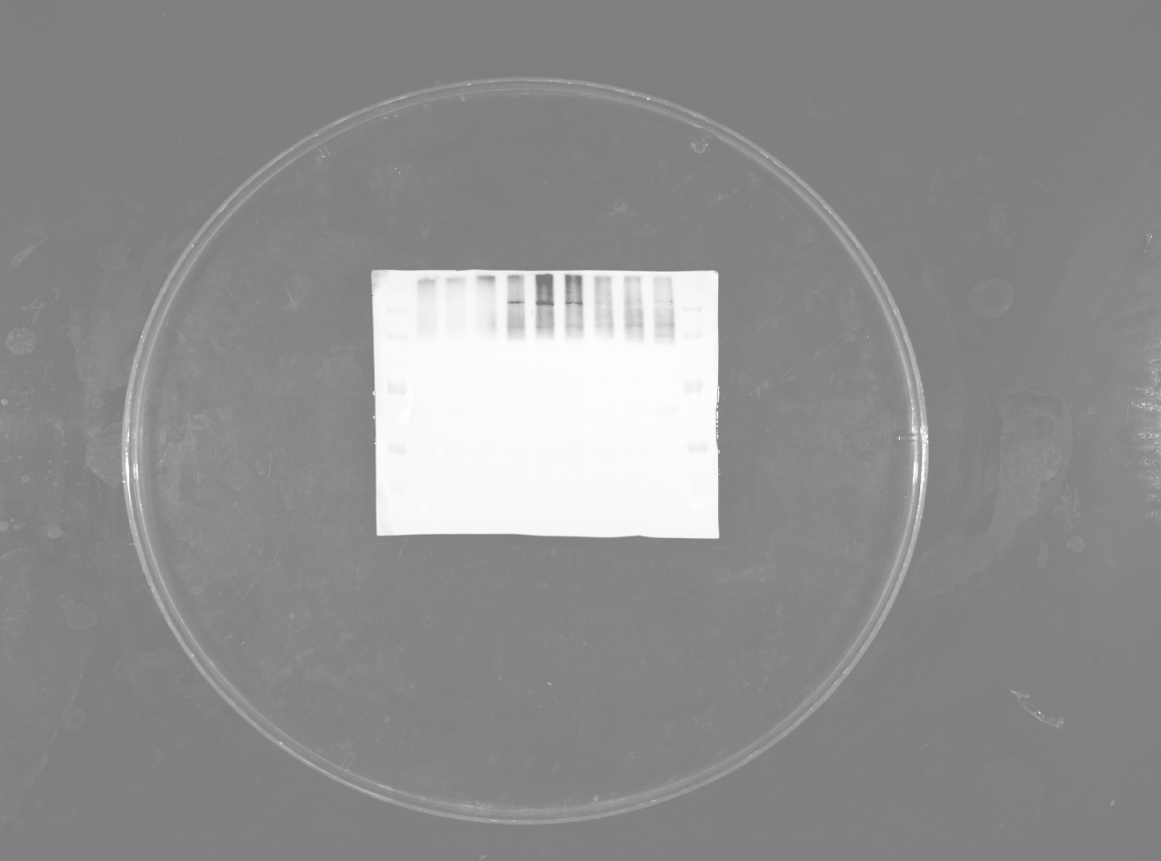


Figure3α-SMA,Vimentin,CollagenⅠ-Gapdh original image


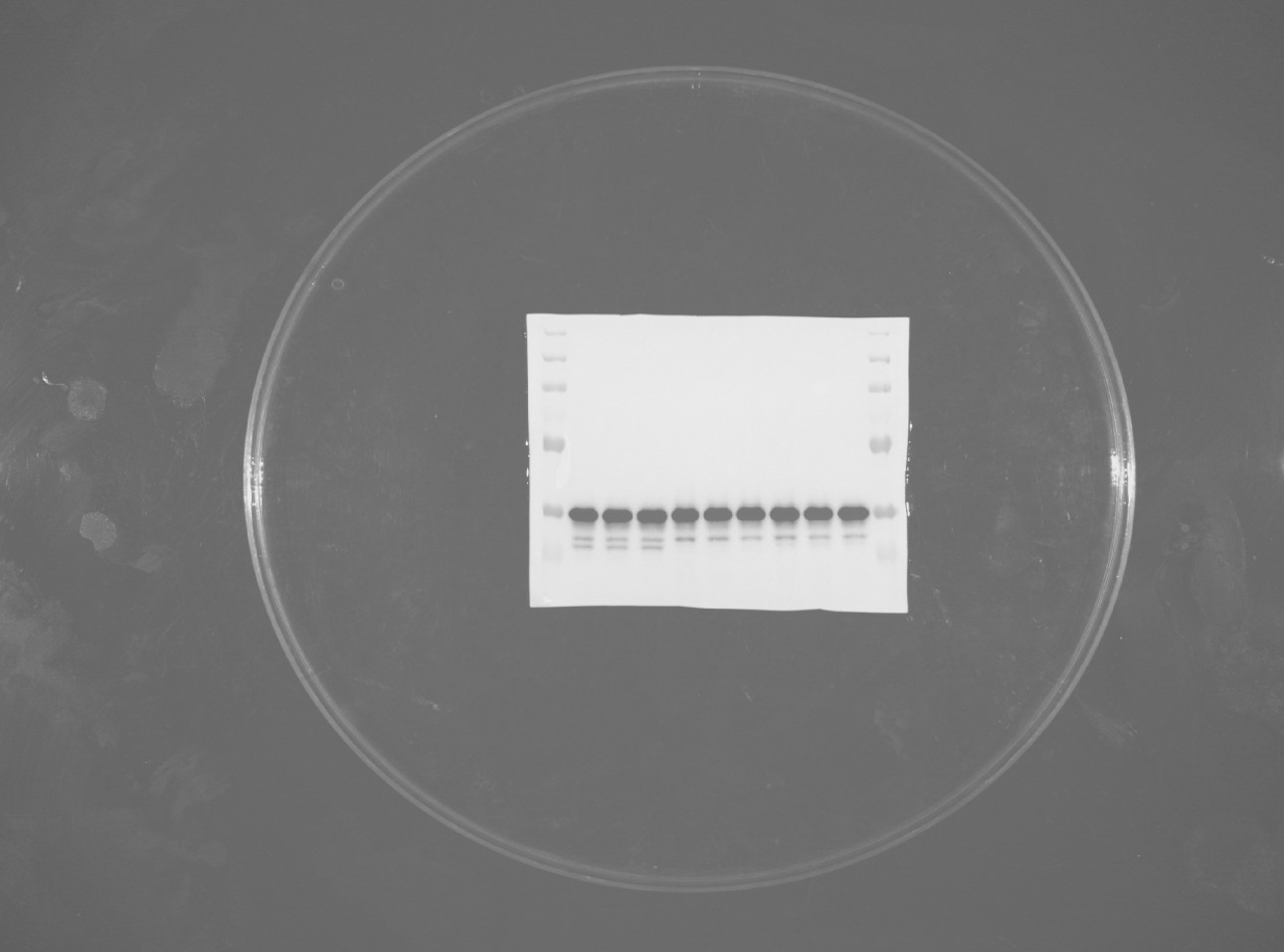


Figure4-B IL-6 original image


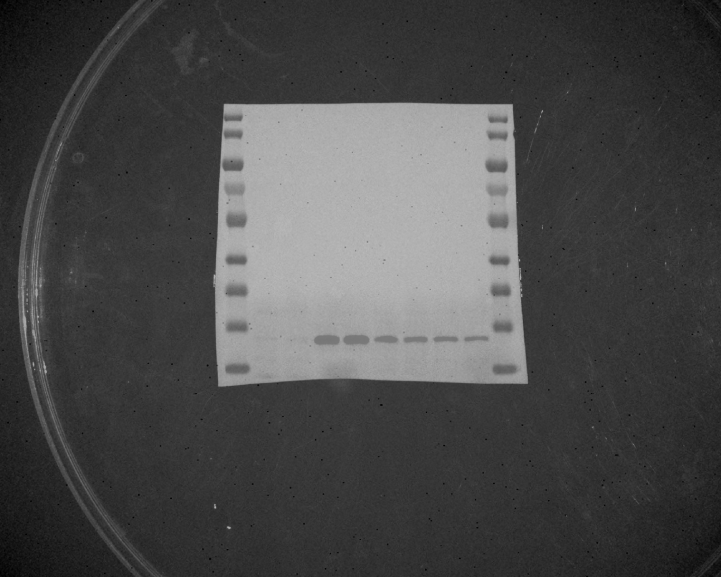


Figure4-B IL-6-Gapdh original image


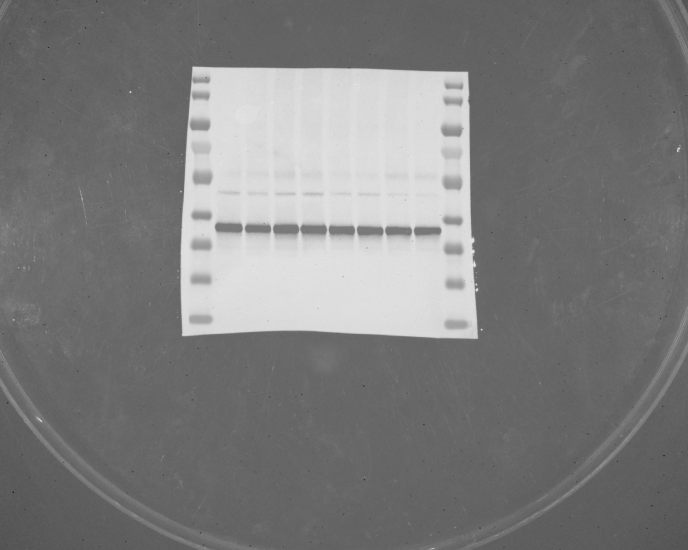


Figure4-B IL-1β original image


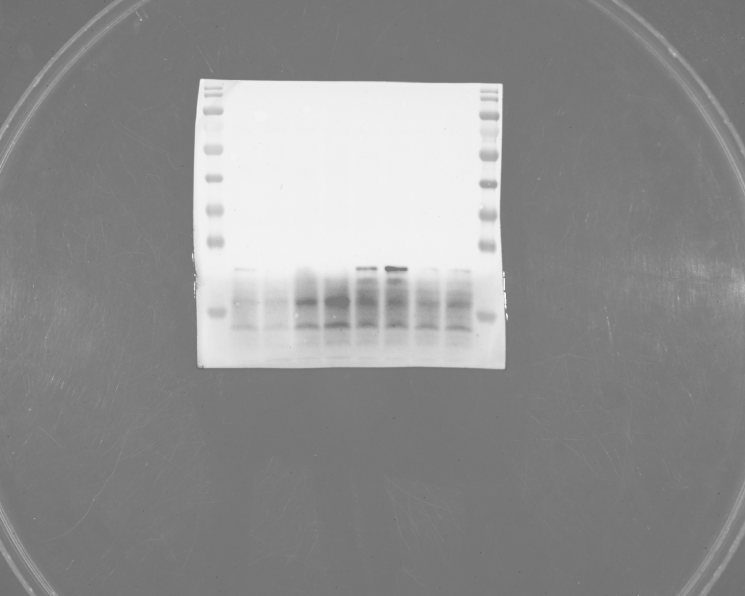


Figure4-B IL-1β-Gapdh original image


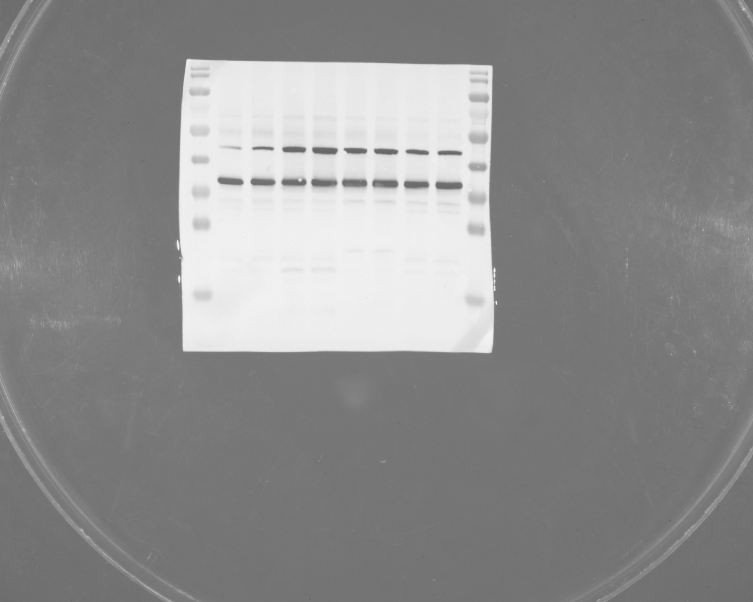


Figure4-B pp65 original image


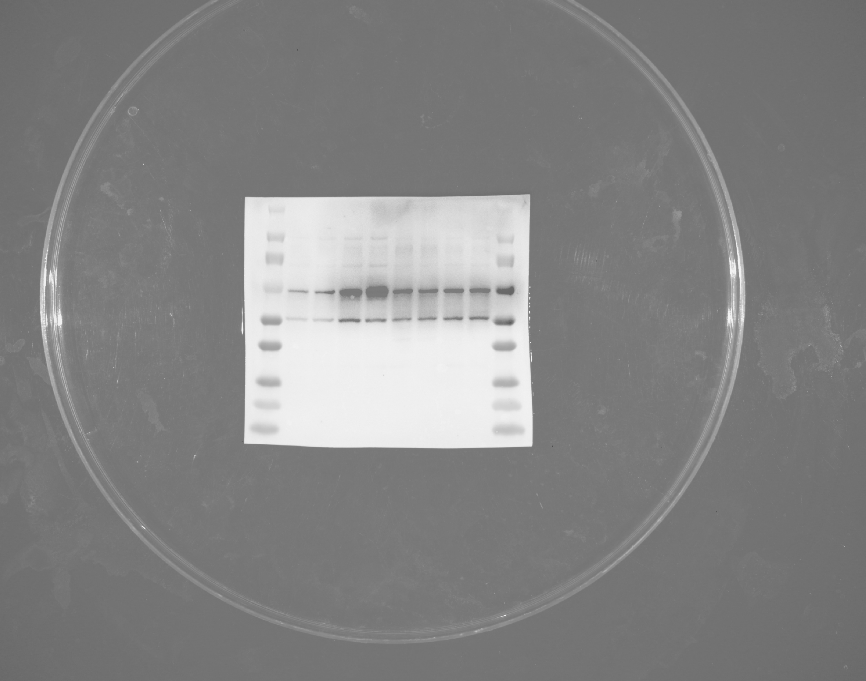


Figure4-B p65 original image


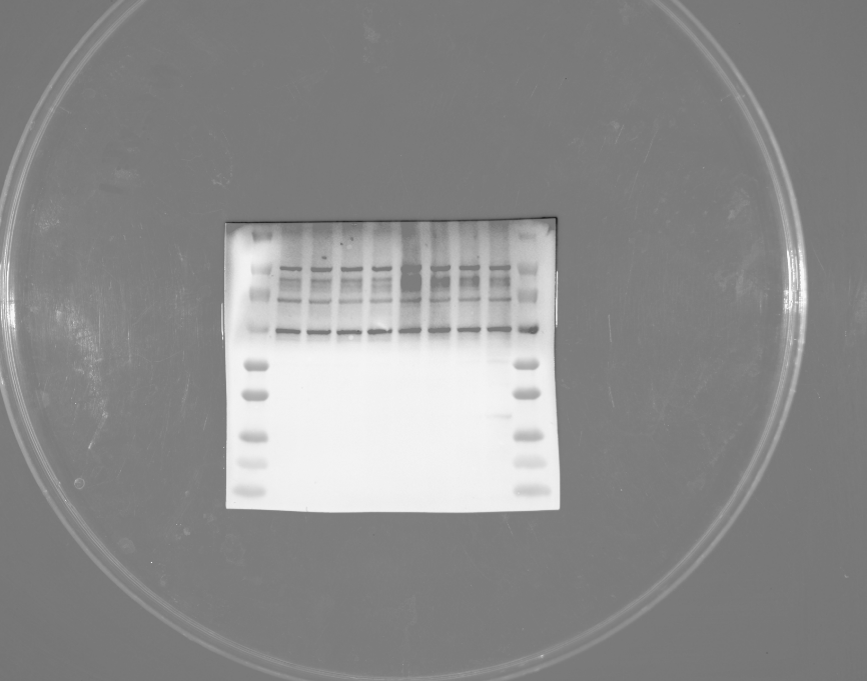


Figure4-B p-ikbα original image


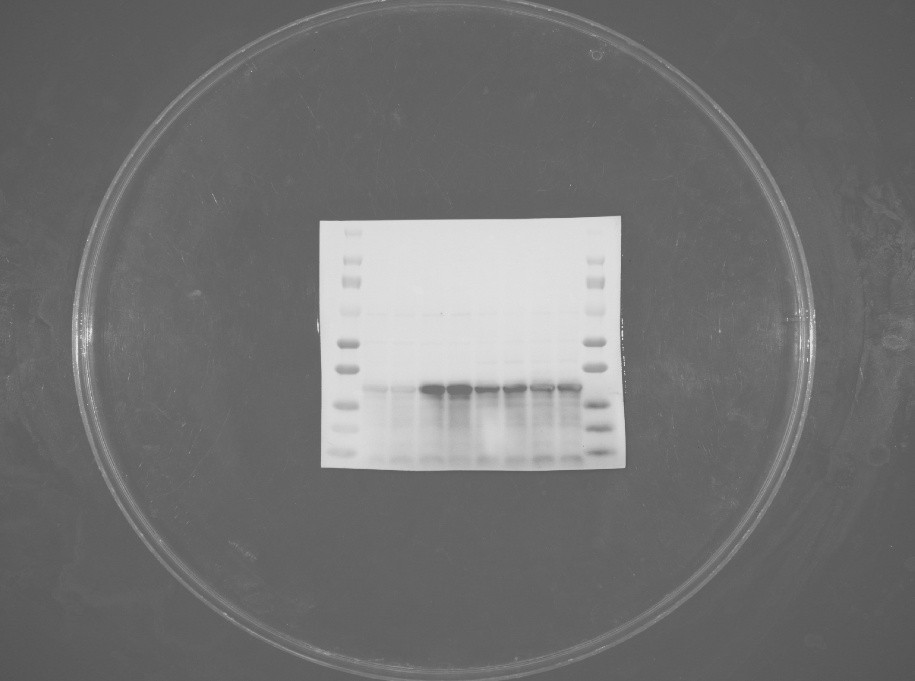


Figure4-B ikbα original image


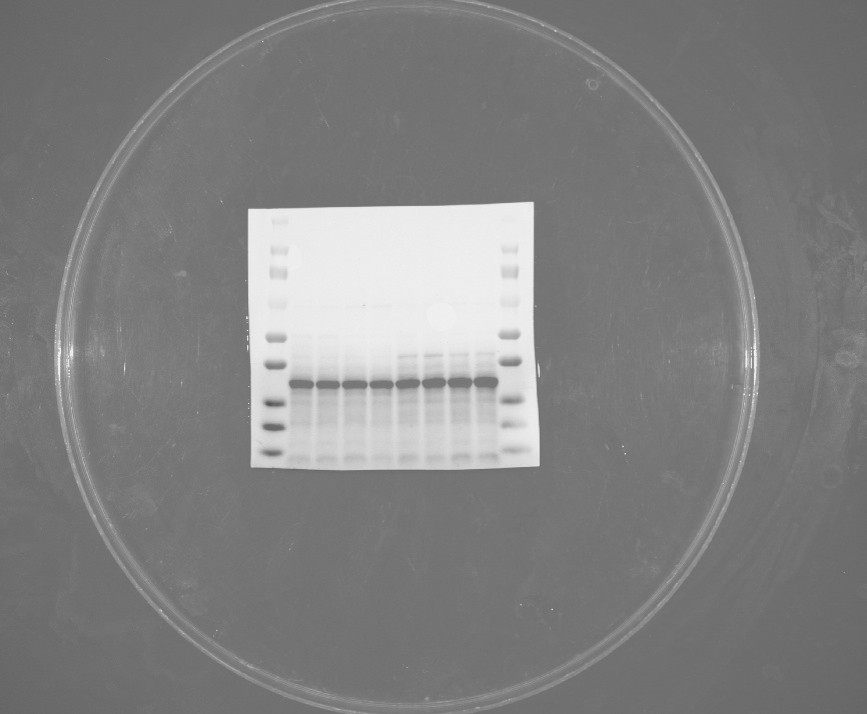


Figure4-B β-actin original image


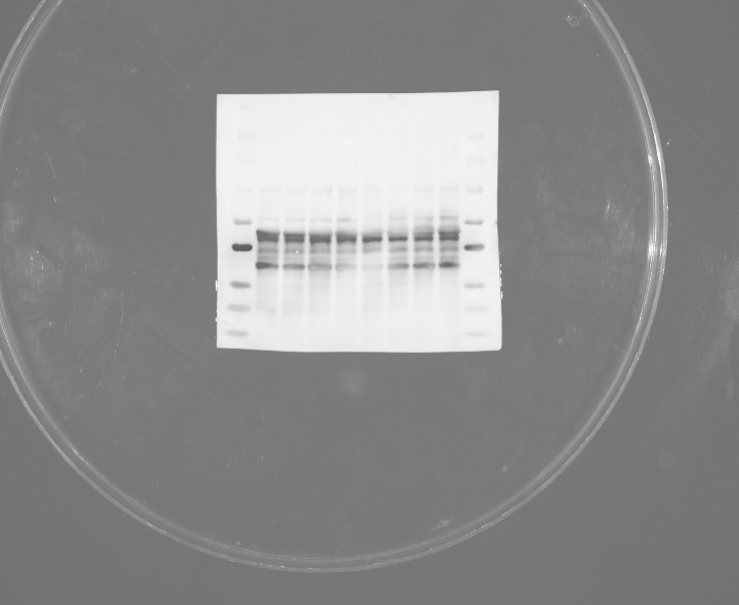


Figure4-D IL-6 original image


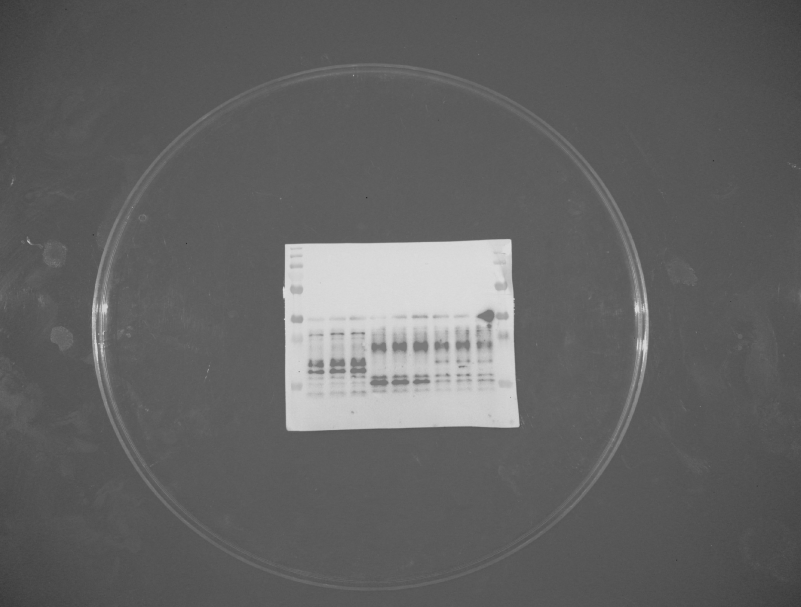


Figure4-D IL-1β original image


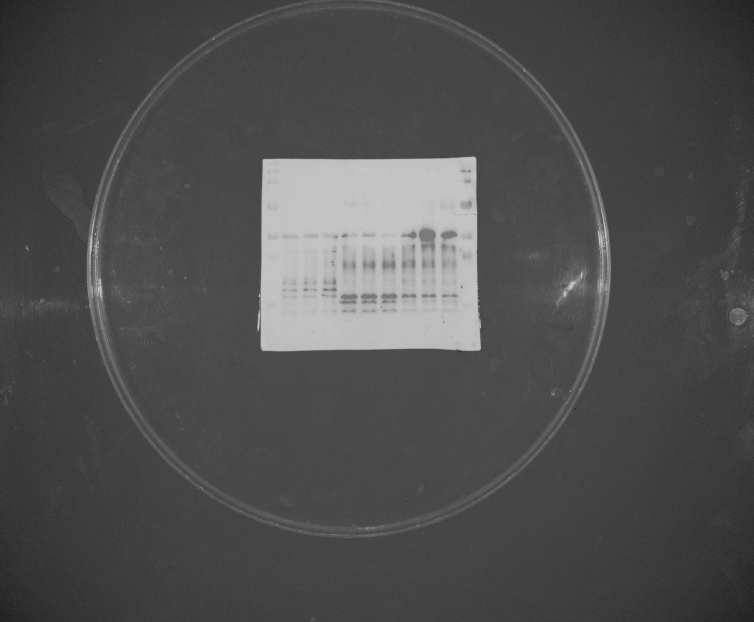


Figure4-D IL-6,IL-1β-Gapdh original image


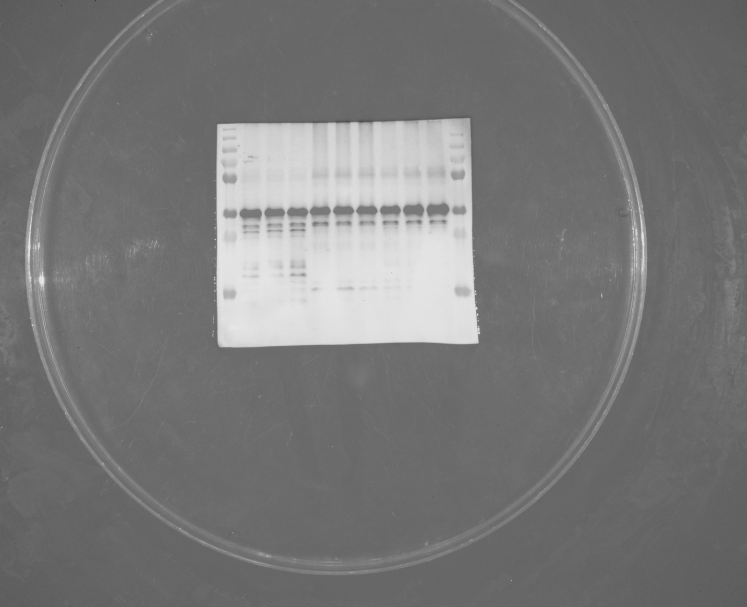


Figure4-D pp65 original image


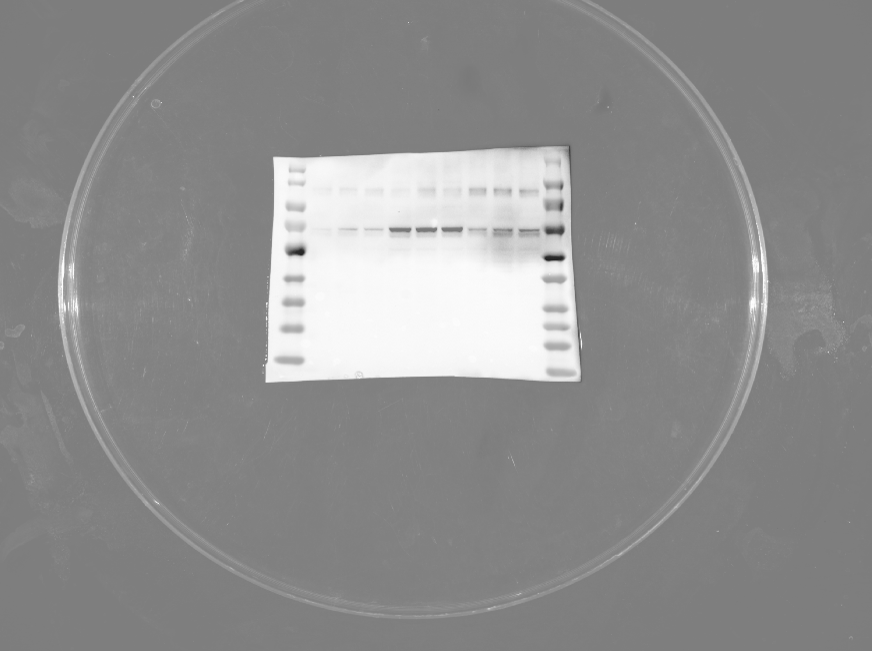


Figure4-D p65 original image


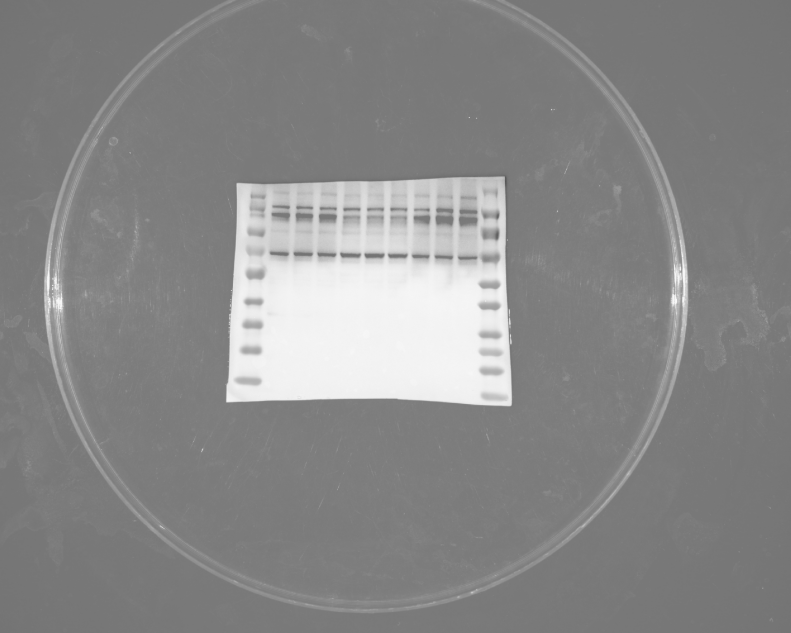


Figure4-D p-ikbα original image


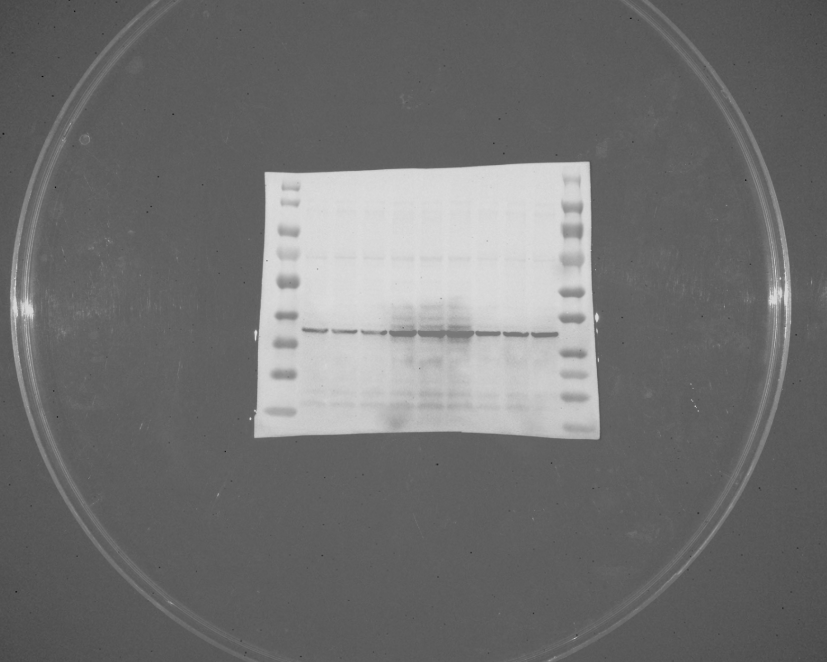


Figure4-D ikbα original image


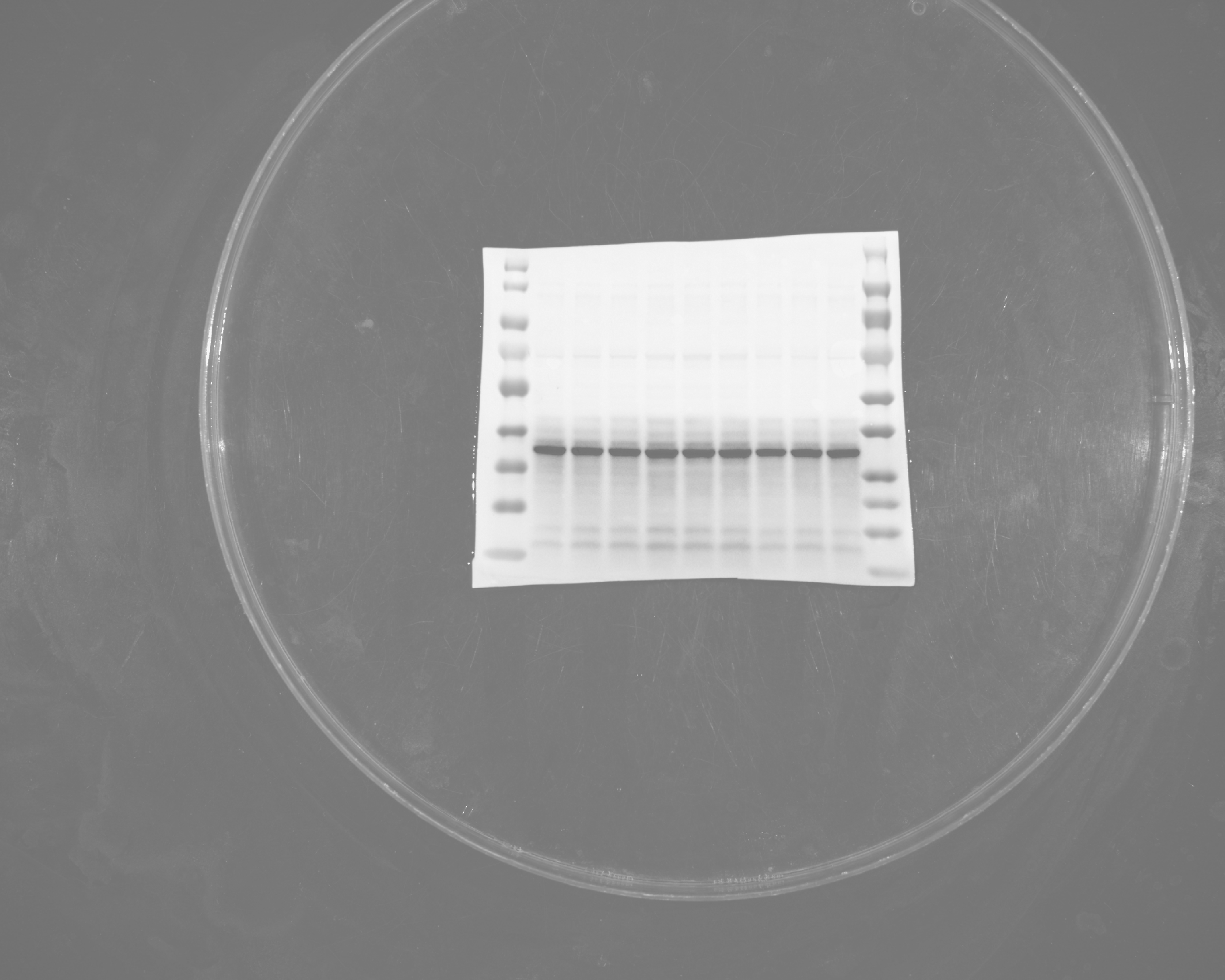


Figure4-D β-actin original image


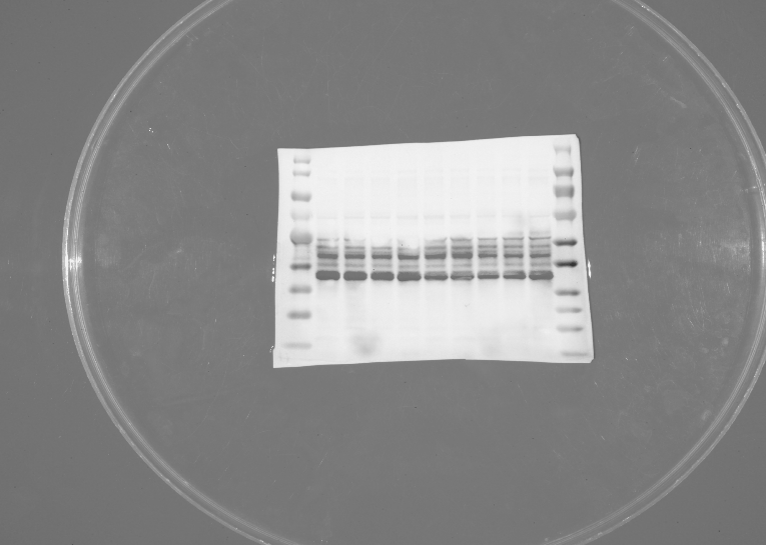


Figure5-B α-SMA original image


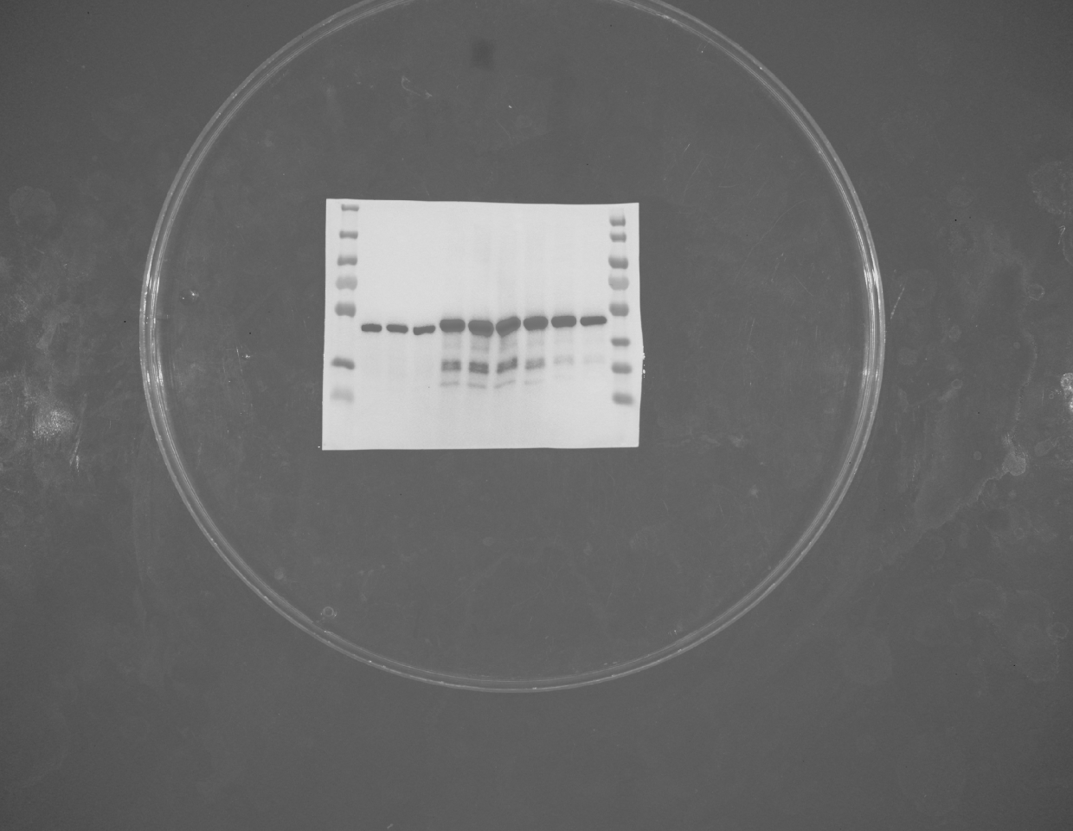


Figure5-B vimentin original image


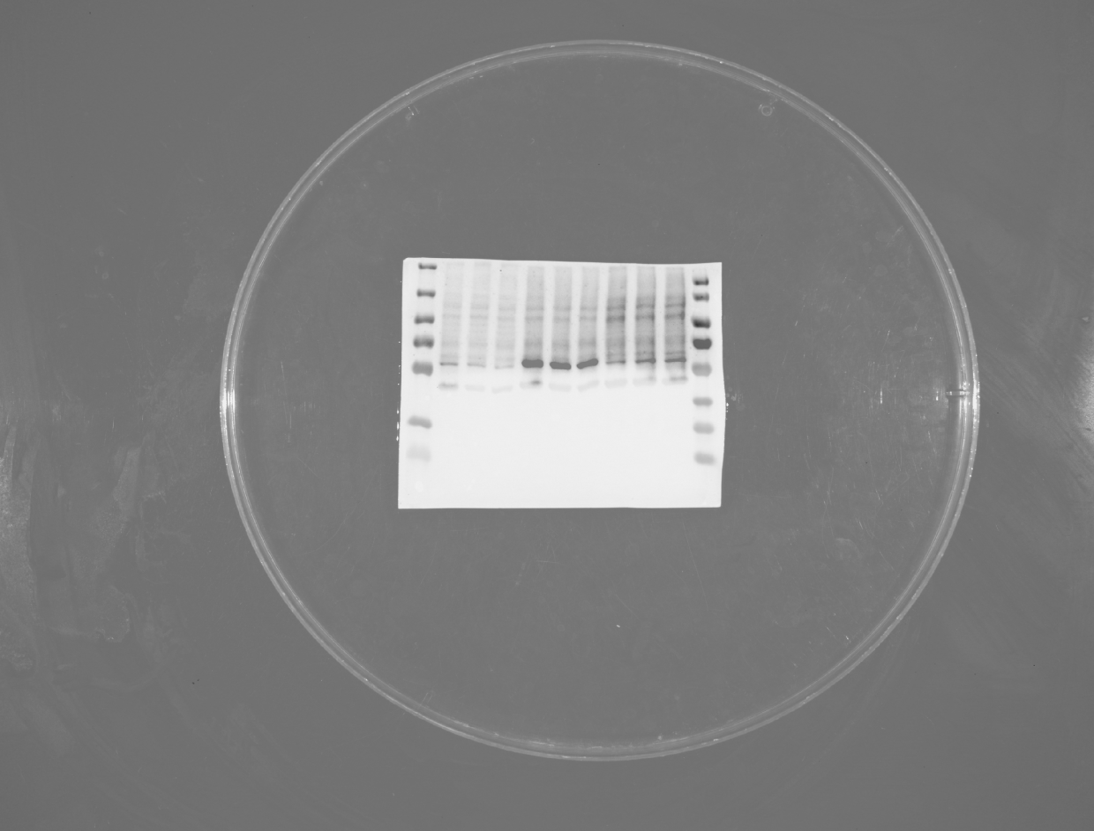


Figure5-B CollagenⅠ original image


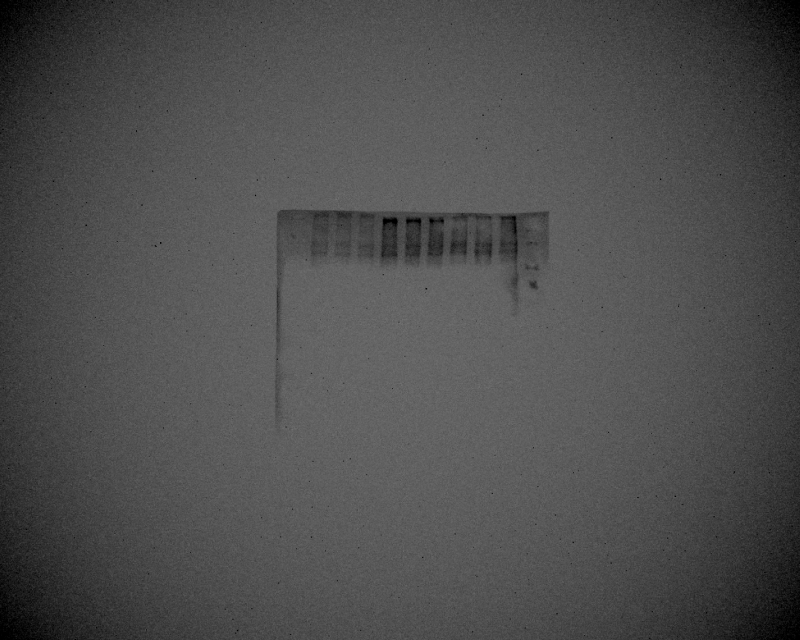


Figure5-B Gapdh original image


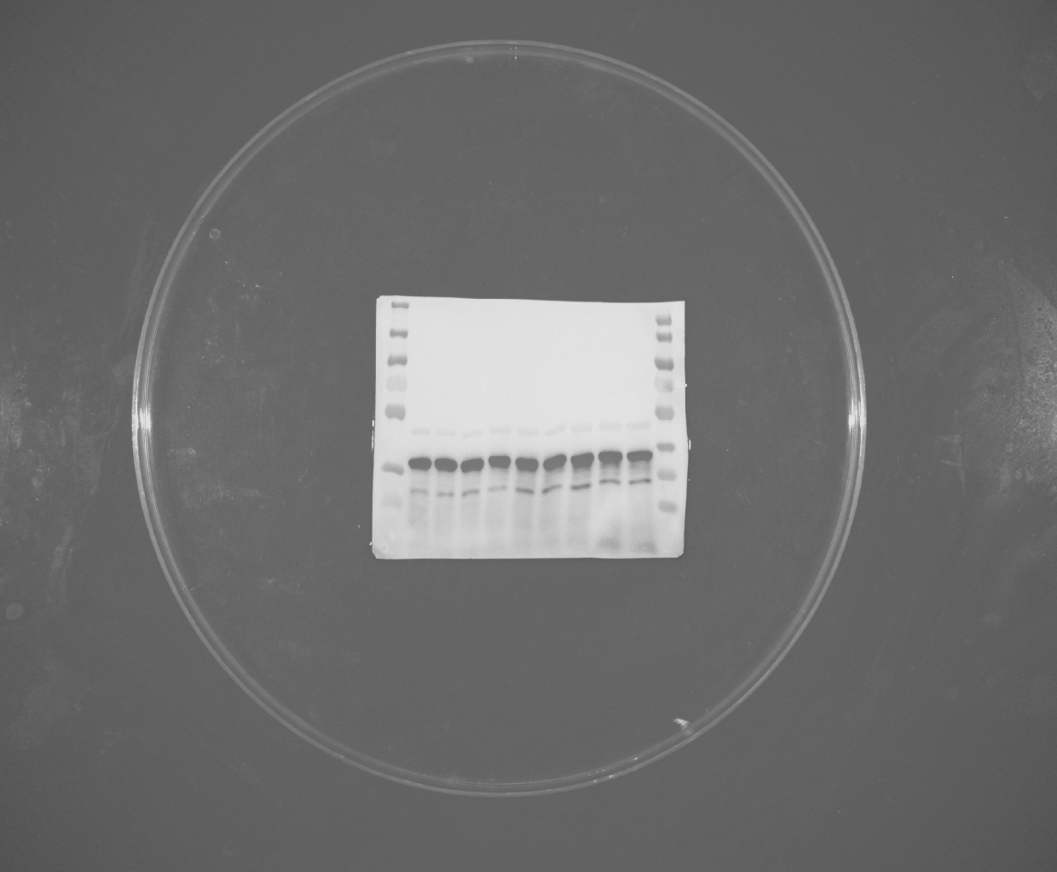


Figure5-B pp65 original image


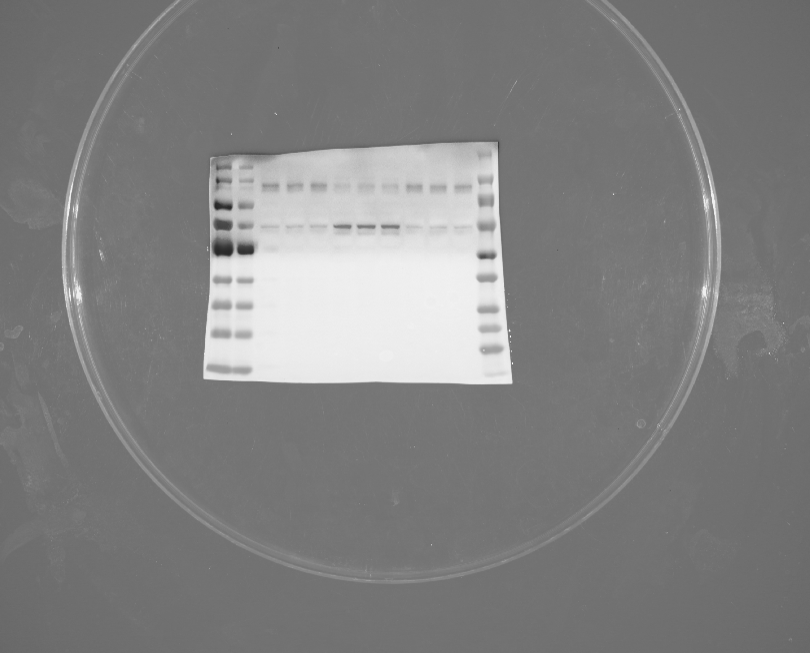


Figure5-B p65 original image


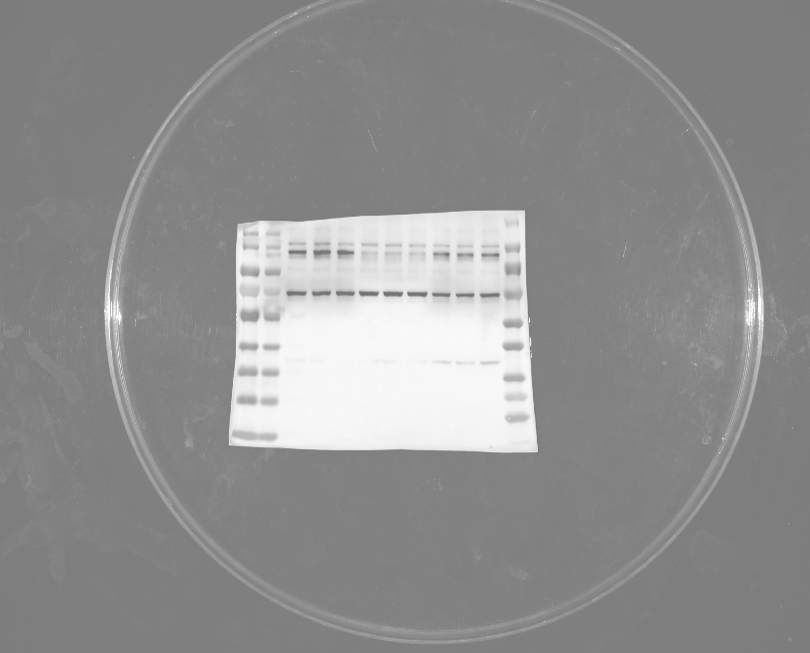


Figure5-B p-ikbα original image


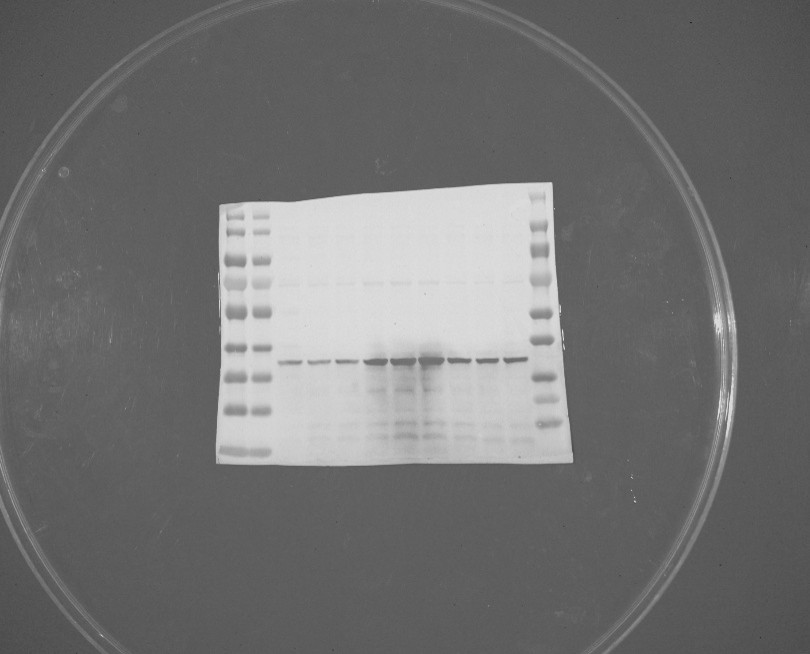


Figure5-B ikbα original image


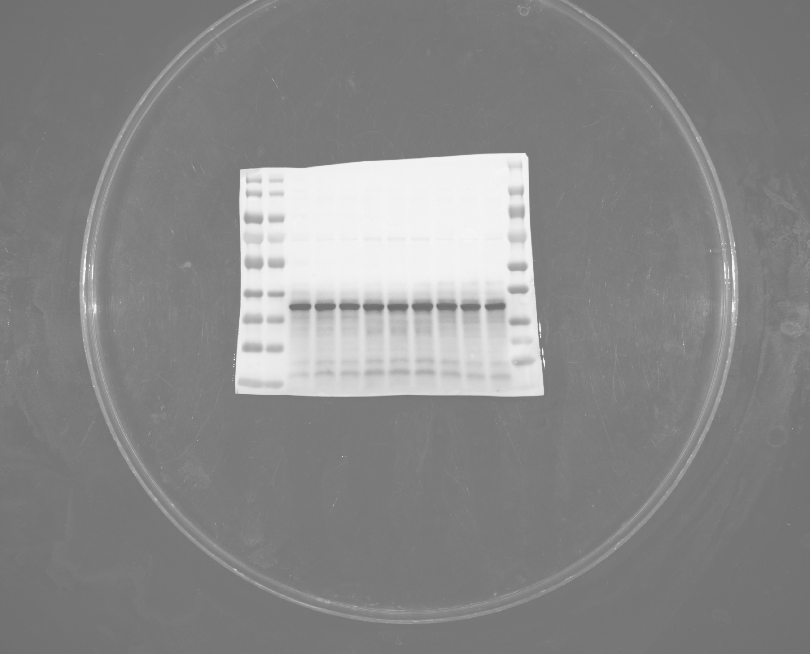


Figure5-B β-actin original image


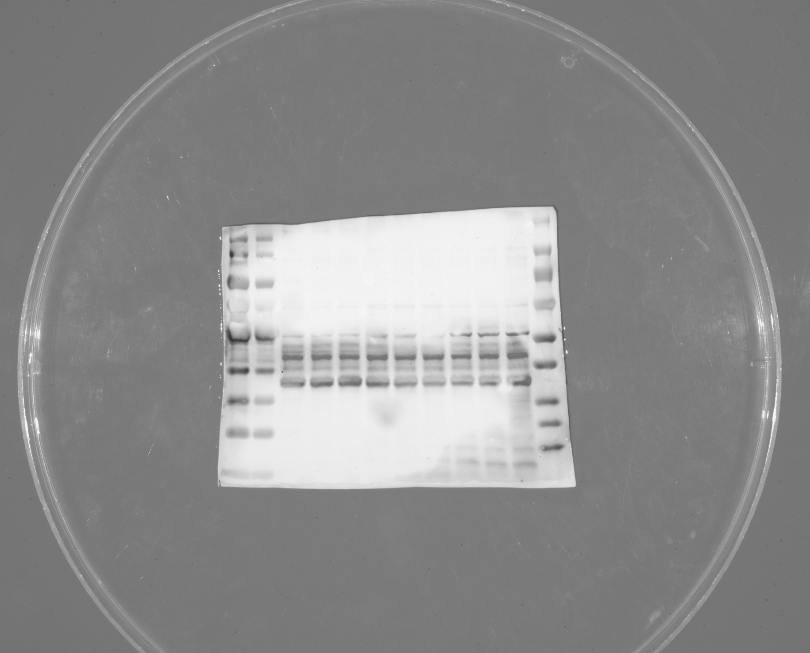


Figure5-E α-SMA original image


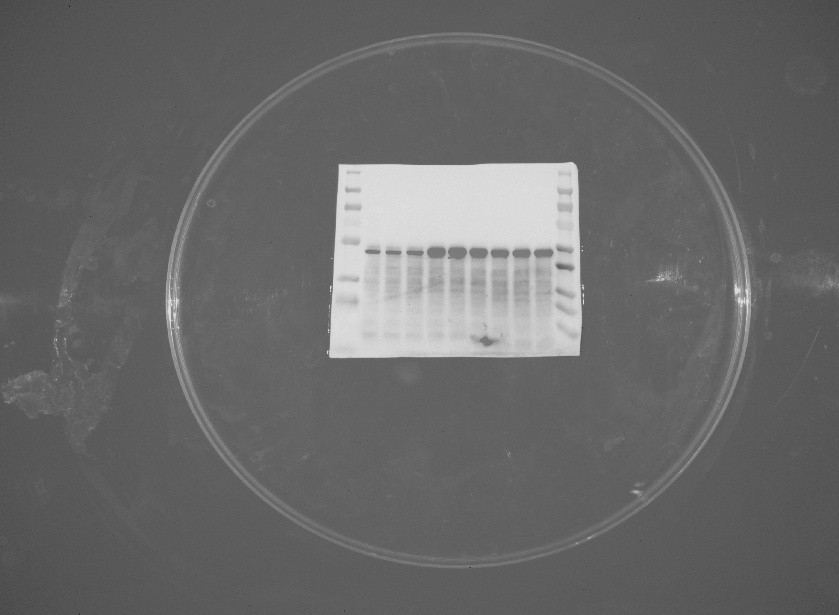


Figure5-E vimentin original image


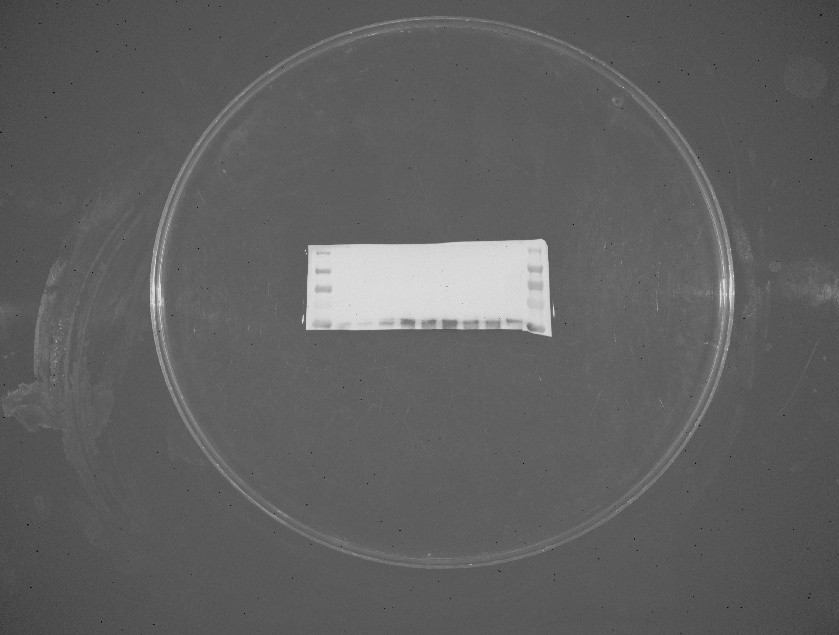


Figure5-E Gapdh original image


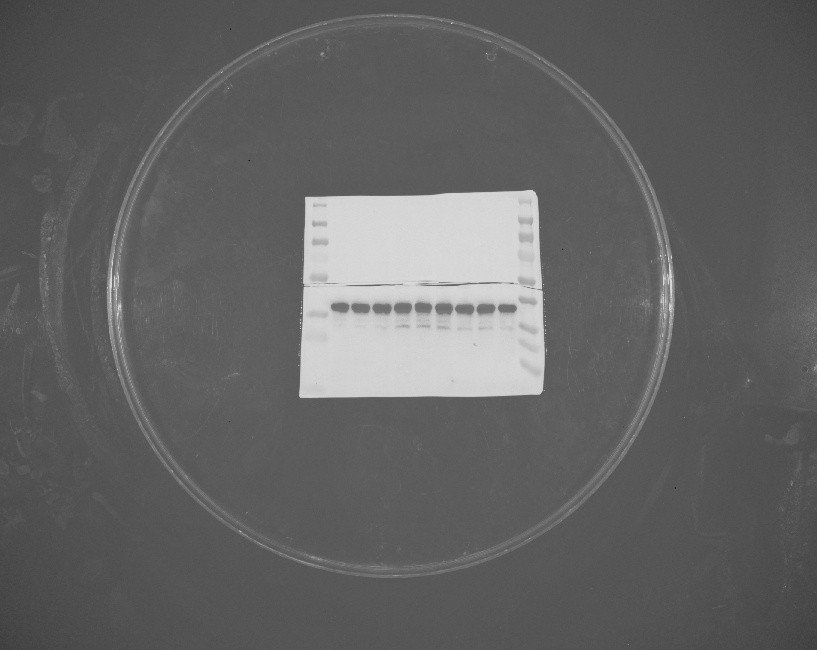


Figure5-E CollagenⅠ original image


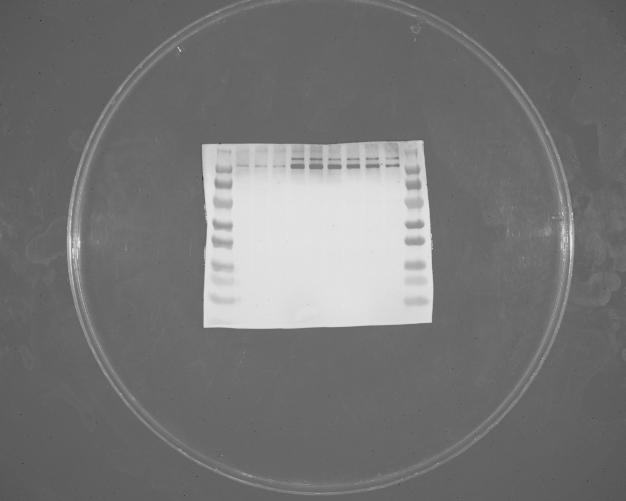


Figure5-E CollagenⅠ-Gapdh original image


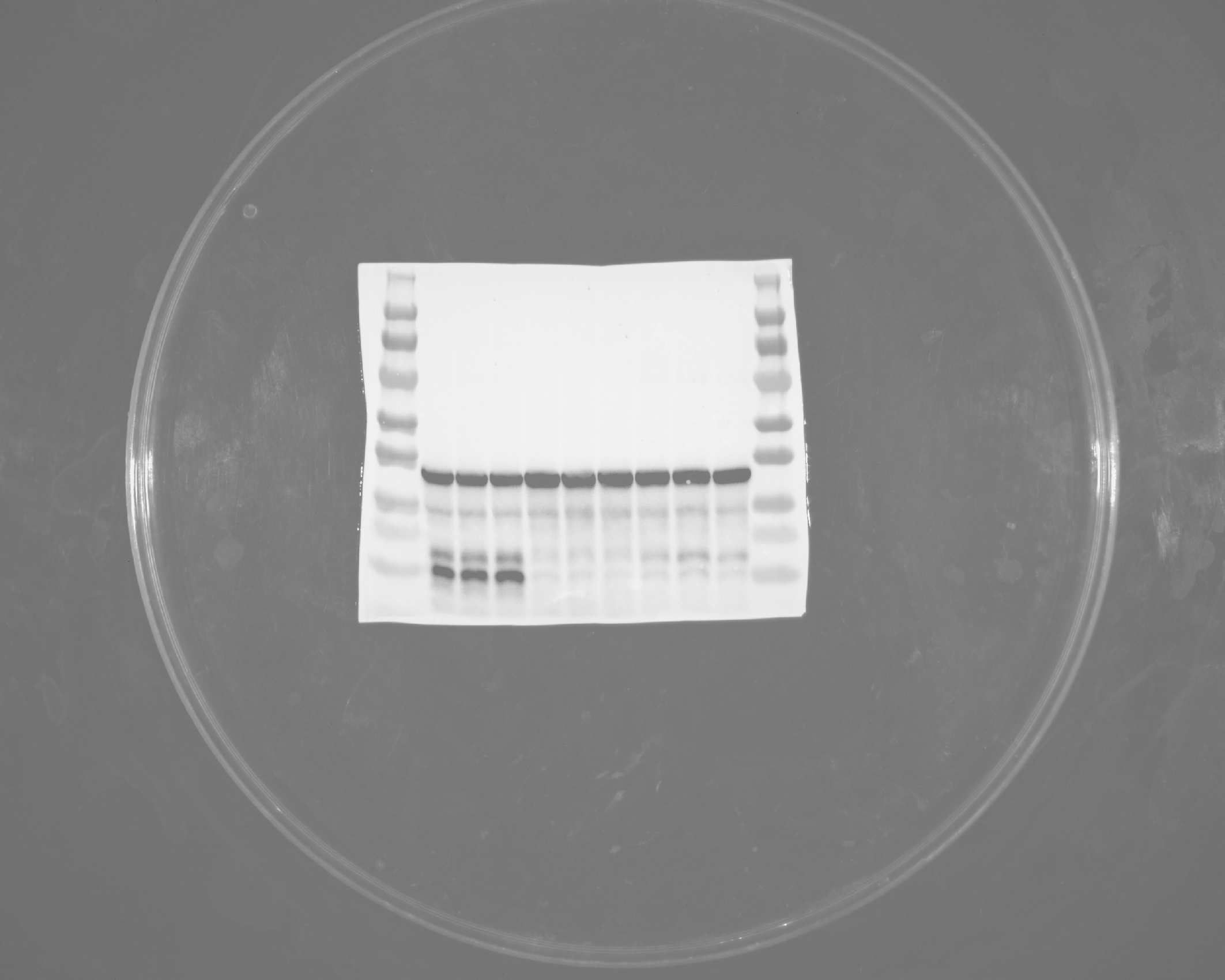

Supplement: Supplementary file 1 — Supplementary Material 1 [file 41598_2026_42540_MOESM1_ESM.docx]
