# Supplementary material for: Targeted deletion of c-kit in TECs attenuates UUO-induced renal fibrosis through NF-κB pathway inhibition
Source: Sci Rep. 2026 Mar 12;16:13227. doi: 10.1038/s41598-026-42540-w (PMC13103321; doi:10.1038/s41598-026-42540-w)
Supplement: Supplementary file 2 — Supplementary Material 2 [file 41598_2026_42540_MOESM2_ESM.docx]

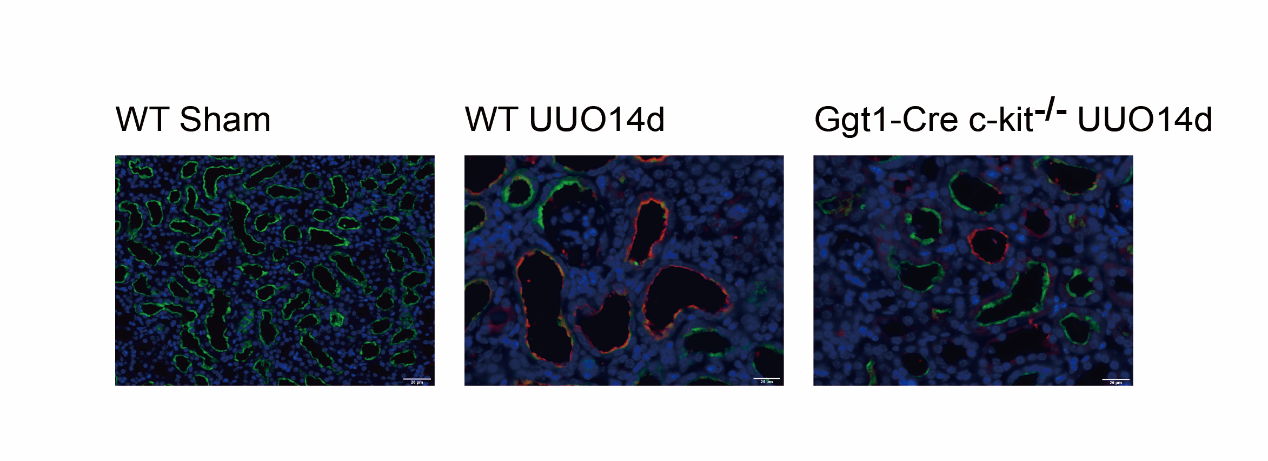
**Supplementary Figure 1. Immunofluorescence co‑staining of Lotus Tetragonolobus Lectin and c‑kit in kidney tissues of WT and Ggt1‑Cre c‑kit⁻/⁺ mice**

Note: Lotus Tetragonolobus Lectin (LTL): green; c‑kit: red; DAPI: blue.
