# Supplementary figures and images for: Targeted deletion of c-kit in TECs attenuates UUO-induced renal fibrosis through NF-κB pathway inhibition
Source: Sci Rep. 2026 Mar 12;16:13227. doi: 10.1038/s41598-026-42540-w (PMC13103321; doi:10.1038/s41598-026-42540-w)

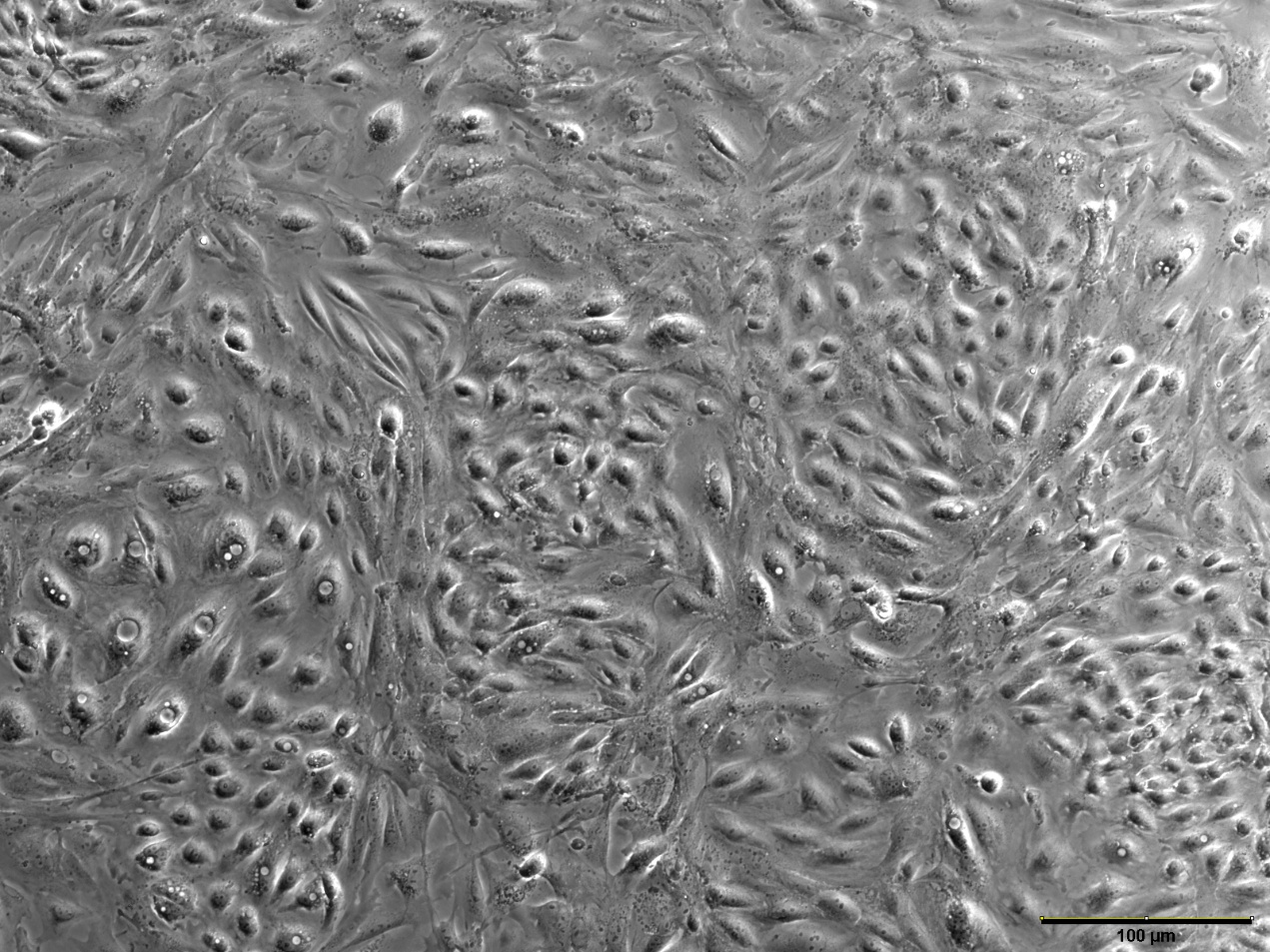


**Supplementary Figure 2. Primary renal tubular epithelial cells (P1 1:2)**

Supplement: Supplementary file 3 — Supplementary Material 3 [file 41598_2026_42540_MOESM3_ESM.docx]
