# Supplementary material for: Targeted deletion of c-kit in TECs attenuates UUO-induced renal fibrosis through NF-κB pathway inhibition
Source: Sci Rep. 2026 Mar 12;16:13227. doi: 10.1038/s41598-026-42540-w (PMC13103321; doi:10.1038/s41598-026-42540-w)
Supplement: Supplementary file 4 — Supplementary Material 4 [file 41598_2026_42540_MOESM4_ESM.docx]

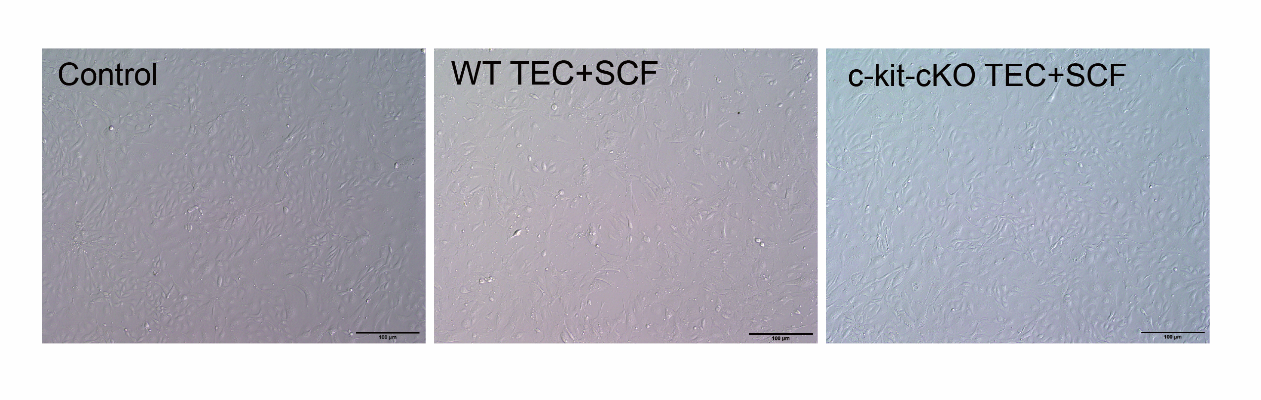


**Supplementary Figure3. Morphology of WT and c-kit cKO primary renal tubular epithelial cells after SCF stimulation.**

Cells in the WT group exhibited a fibrotic phenotypic transformation, while no significant changes were observed in the c-kit cKO compared to the control (10×, n=3).
