# Supplementary material for: Targeted deletion of c-kit in TECs attenuates UUO-induced renal fibrosis through NF-κB pathway inhibition
Source: Sci Rep. 2026 Mar 12;16:13227. doi: 10.1038/s41598-026-42540-w (PMC13103321; doi:10.1038/s41598-026-42540-w)
Supplement: Supplementary file 5 — Supplementary Material 5 [file 41598_2026_42540_MOESM5_ESM.docx]

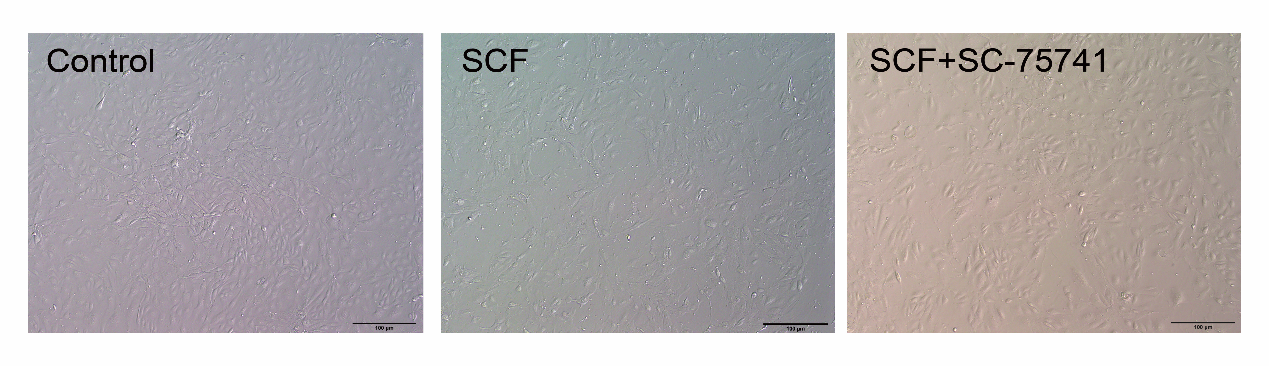


**Supplementary Figure4. Primary TECs treated with SCF and SCF + NF-κB inhibitor SC-75741**. Fibrotic phenotypic transformation was observed in the SCF-treated group (10×, n=3).
